# Supplementary material for: Development of a peer-led, network mapping intervention to improve the health of individuals with severe mental illnesses: protocol for a pilot study
Source: BMJ Open. 2019 Jun 29;9(6):e023768. doi: 10.1136/bmjopen-2018-023768 (PMC6609054; doi:10.1136/bmjopen-2018-023768)
Supplement: Author's manuscript [file bmjopen-2018-023768.draft_revisions.pdf]

BMJ Open is committed to open peer review. As part of this commitment we make the peer review history of every article we publish publicly available.

When an article is published we post the peer reviewers' comments and the authors' responses online. We also post the versions of the paper that were used during peer review. These are the versions that the peer review comments apply to.

The versions of the paper that follow are the versions that were submitted during the peer review process. They are not the versions of record or the final published versions. They should not be cited or distributed as the published version of this manuscript.

BMJ Open is an open access journal and the full, final, typeset and author-corrected version of record of the manuscript is available on our site with no access controls, subscription charges or pay-per-view fees (http://bmjopen.bmj.com).

If you have any questions on BMJ Open's open peer review process please email info.bmjopen@bmj.com

BMJ Open

Managing transition from secondary mental to primary health care using peer-delivered well-being network mapping: protocol for a pilot study

Journal:	*BMJ Open*
Manuscript ID	bmjopen-2018-023768
Article Type:	Protocol
Date Submitted by the Author:	23-Apr-2018

Complete List of Authors:	Collom, Jennifer; King's College London, Institute of Psychiatry, Psychology and Neuroscience<br>Davidson, Jonathan ; Institute of Psychiatry, Psychology and Neuroscience, Health Service and Population Research Department<br>Sweet, Daryl ; McPin Foundation<br>Gillard, Steve; St George's University of London, Division of Population Health Sciences and Education<br>Pinfold, Vanessa; McPin Foundation,<br>Henderson, Claire; King's College London, ; South London and Maudsley NHS Foundation Trust,

Keywords:	Physical health, Peer support, Well-being network mapping, Intervention, Pilot, Protocol

SCHOLARONE™ Manuscripts

For peer review only

Managing transition from secondary mental to primary health care using peer-delivered well-being network mapping: protocol for a pilot study

Authors

Jennifer Collom[1] MSc

Jonathan Davidson[1] MA, MSc

Daryl Sweet[2]

Steve Gillard[3] PhD

Vanessa Pinfold[2] PhD

Claire Henderson[1] MRCPsych, PhD

Affiliations:

1. Health Service and Population Research Department, King's College London, Institute of Psychiatry, Psychology and Neuroscience, London, UK.
2. McPin Foundation, London, UK.
3. Population Health Research Institute, St Georges University of London, London, UK.

Correspondence to: Jennifer Collom, Health Service and Population Research Department, King's College London, Institute of Psychiatry, Psychology and Neuroscience, 16 De Crespigny Park, London, UK, SE5 8AF. Jennifer.Collom@kcl.ac.uk 0207 848 5087

Word Count: 3844

For peer review only

ABSTRACT

Introduction

Adults with Severe Mental Illness (SMI) have reduced life expectancy and many have comorbid physical health conditions. Primary care providers are experiencing increased demands to care for people with SMI. Barriers to accessing physical health care have been identified which negatively affect quality of care. We propose that Peer Support Workers (PSWs) could deliver an intervention to service users to promote their physical health by drawing on existing social support.

The objective of this research is to pilot a PSW led intervention, including Personal well-being network mapping, to improve access to primary care for physical health needs. We aim to deliver the intervention in two boroughs; create a theory of change; and to inform the design of a future larger scale trial of the intervention.

Methods and analysis

Participants will be recruited via community mental health teams in two London boroughs (n=24). Each participant will be offered a 6 session intervention. Quantitative data will be collected pre and post intervention (at 4 month follow up). Qualitative interviews will be conducted with PSWs after completion of the intervention and with participants at a 4 month follow up. Some intervention sessions will be observed by a member of the research team. This is a pilot study with a small sample aiming to assess acceptability and feasibility of an intervention. This study is strengthened by its potential clinical value and origin in previous research where service users engaged with well-being network mapping.

Ethics and dissemination

This study has been approved by the London – Chelsea Regional Ethics Committee (ref: 17/LO/0585). The findings will be disseminated to participants, the NHS trusts that we recruited from, primary care mental health leads, commissioners and in peer-reviewed journals and academic conferences.

Keywords

Physical health, peer support, well-being network mapping, intervention, pilot, protocol.

ARTICLE SUMMARY

Strengths and limitations of this study

For peer review only

- The research will employ a mixed methods design; the qualitative data collected from participants and PSWs will aid the development of the intervention.
- The study will inform a larger scale research study into the intervention and in turn, could inform the development of a new approach to physical healthcare for people with SMI.
- Robust statistical analysis cannot be performed on the data due to the small sample size and no objective physical health measurements will be collected; this study does not aim to pilot collection of physical health data.
- The qualitative data collection employed by this study can be viewed as non-generalizable.

For peer review only

INTRODUCTION

People with severe mental illness (SMI) have greatly reduced life expectancy compared to the rest of the population, of 15-20 years depending on gender and diagnosis (1). High rates of smoking, obesity and low levels of exercise contribute to this (2, 3). Engagement with primary care practitioners and facilities for health promotion (such as leisure facilities, smoking cessation groups, social activities) is increasingly important as many people with SMI have been discharged from secondary care mental health services, and care pathways have been redesigned to accelerate discharge after both referral and re-referral from primary care. Research published in 2012 found a high proportion of people with SMI are supported solely by a GP (31%) or with minimal secondary care input (4). This number is increasing as thresholds for admission to secondary mental health services are raised and more people discharged back to primary care (5).

However, this group has generally poor access to primary care for physical health needs. Primary care within current service configurations is not best placed to absorb increasing numbers of people with severe mental illness, discharged from secondary provision (4) and there is wide variation in the extent to which primary care is proactive in ensuring access to care for physical health problems (6).

Similarly to people with SMI, people with learning disabilities experience poorer physical health and lower life expectancies than the general population (7). However, general practitioners respond to these groups differently. The Royal College of General Practitioners recommends extended appointment times, of 30 minutes, for people with learning disabilities (8). Currently, specific provision is not made for people with SMI.

Attitudes to people with severe mental illnesses may create barriers to responses to physical health complaints. In the USA, Corrigan and colleagues have found a relationship between attitudes and treatment intentions among mental health and primary care professionals working in the US Veteran's Health Administration. Path analyses showed participants who endorsed stigmatizing characteristics of a male patient with schizophrenia described in a vignette were more likely to believe the patient would not adhere to treatment; as a result, they were less likely to refer to a specialist or refill his prescription (9). Some participants in qualitative research carried out in a

For peer review only - http://bmjopen.bmj.com/site/about/guidelines.xhtml

London emergency department reported that they avoid people who are experiencing symptoms of mental illness due to fear of violence, which may also adversely affect quality of care (10). Fear of patients with substance abuse problems has also been expressed by district nurses, with the consequent risk of suboptimal care (11).

There is also evidence from the US that family physicians are less likely to believe that patients with previous depression have serious medical conditions causing physical symptoms, leading to a greater reluctance to carry out investigations for such symptoms (12). This may reflect the misattribution of physical symptoms to pre-existing mental illness (13), a phenomenon known as 'diagnostic overshadowing' (14). A qualitative interview study of emergency department nurses and doctors (10) revealed that this is a fairly well recognised problem that can lead to adverse consequences varying from delay in treatment to death. Research has outlined how many GPs: are less keen on supporting people with SMI than other medical conditions (15); lack specialist knowledge and skills (16); and have clear ideas that their role should be limited to physical health checks and providing medication (17, 18).

Supported access and empowerment are thus needed for improved physical health care in this group. These functions may be carried out by care co-ordinators for those in secondary care, however caseload sizes may not permit the time needed to focus on physical health and those whose mental health is fairly stable are likely to be discharged to primary care (19). Evidence from a number of trials reviewed recently suggests that some practitioner roles can be performed by trained and supervised peer support workers leading to similar outcomes as when they are undertaken by mental health staff (20). There is also a potential longer term advantages of employing peer workers in this role; it creates a rung on the career ladder for the peer, which is also visible to the person in receipt of their support, encouraging recipients to in turn become a peer supporter or take on other similar roles (21, 22).

This project draws on peer-led (23, 24) and peer co-facilitated (25) interventions in the US to improve self-management of physical health and increase uptake of primary care services for physical health conditions, and on UK research exploring social networks and their potential resources to support people with SMI. The first care navigator study drew upon existing models of social network mapping but added location and activity dimensions to social contacts, having found that all three had meaning to people with SMI and assisted them in managing their well-being (26). The second is the Connecting People project funded by the National School of Social Care Research (27). The third builds on the Community Network Research study (28); this recruited through primary care and has developed a pilot intervention using a co-production process with multiple stakeholders within the London Borough of Hounslow. These projects seek

For peer review only

to address social isolation, by encouraging network development or enhancement using workers to empower and signpost individuals to opportunities locally to build connections to people, places and activities. This preliminary work has found some promising building blocks in the form of personal well-being networks; however, it employed a cross sectional design and as yet has not been taken into an intervention. The current study will address this, by piloting personal well-being network mapping as part of an intervention.

Well-being network mapping was developed by the McPin Foundation (29) following their study of social networks and how they relate to personal well-being (30). Support networks are made up of social contacts, and recovery is linked to meaningful activities, identity and sense of purpose in life, empowerment, hope and connectedness. Further, community places and spaces constitute the environments in which people look after their health and well-being and build social networks. Well-being network mapping is asset based, strengths focused and through structured conversations may facilitate engagement and planning to address identified health and social care needs. The use of this tool will allow peers to assess the client's relationship with primary care, in the context of other health related behaviours and relationships which can promote or adversely affect health. The PSW will work with their client to utilise the connections established using the map with the aim of improving well-being in a sustained way, with a focus on access for physical health needs.

The project will adopt a theory of change approach (31, 32). This is a method for developing a framework for delivering change, and explaining the mechanisms for doing so. It covers assumptions, inputs, and mechanisms and suggests appropriate outcomes based on underlying processes in order to better understand what is involved in achieving sustained long term change. Previous work on well-being network mapping has not employed this approach. It is hoped that the theory of change will improve current understanding of how people move from identifying resources and barriers in their networks, to making positive changes to their physical health and access to healthcare.

Aims and objectives

The aims of the project are:

1. To develop a localised model for a peer well-being network mapping intervention in two boroughs.
2. To produce a theory of change, mapping out assumptions, inputs, mechanisms and outcomes.

For peer review only

3. To deliver the intervention in two boroughs and assess impact on people with SMI and the peer support workers.
4. To inform the development of a future feasibility trial to assess feasibility of a pragmatic trial of the effectiveness of the intervention with respect to access to primary care for physical health needs.

METHODS AND ANALYSIS

Design

This study will pilot an intervention to improve physical healthcare utilising well-being network mapping delivered by PSWs. The intervention addresses barriers to accessing physical healthcare and employs behavioural activation through goal setting for people with SMI. All participants will receive the intervention.

Recruitment

A member of the research team will approach clinical staff at community mental health team centres. They will be informed about the research, the inclusion/exclusion criteria, and given an information script. This will provide clinical staff with details of the study to relay to possible participants. When clinical staff receive agreement from a service user, a meeting will be organised; during which, a researcher will provide a patient information sheet (PIS) and answer any questions. Participants will be offered a period of time, usually at least 24 hours, to consider their involvement in the study according to IRAS guidance (42), before providing consent to participant in the study.

Sample size

For preliminary feasibility testing and receipt of the intervention, participants (n = 24) will be recruited via opportunity sampling.

Inclusion and exclusion criteria

The following inclusion criteria will be used: (1) approaching discharge from secondary mental health services, (2) at risk of deterioration in physical health due to poor access to primary care,

For peer review only

(3) demonstrated failure to organise/attend primary care appointments for co-morbid physical health difficulties, (4) aged 18 or over.

People will not be eligible to take part in the study if they: (1) lack capacity to provide informed consent, (2) possess a high risk of harm to others, (3) are unable to communicate in English, (4) are currently an inpatient on a psychiatric hospital and (5) nonattendance of discharge planning meeting with subsequent discharge.

The intervention

Peer support workers: PSWs (n=6) were recruited from within existing peer support services in the two London boroughs (three and four PSWs respectively). The PSWs were paid to take on the peer well-being role. We aimed that each PSW will work with up to 4 clients over a 6 month period. Peer support workers were trained by the McPin Foundation and ENRICH, a programme of research to develop, pilot and trial a peer worker intervention. The McPin Foundation conducted the original personal well-being network study (26) and has implemented this tool in Hounslow. PSWs will be supported with use of the well-being mapping tool through regular supervision with the research team and their line manager.

Well-being network mapping: The therapeutic well-being network tool was collaboratively adapted by peer support workers and the research team. The tool aims to provide support for people who have been discharged form secondary mental healthcare and are not well engaged with primary care for physical health needs.

Over six sessions, the intervention aims to cover the following topics: engagement of the client with the peer and the intervention; mapping of the client's network, including people and services which contribute positively, negatively, or both, to the client's well-being; goal setting for physical health and planning; a visit to a primary care service e.g. their GP's surgery, dentist, or sexual health clinic, to which the client may or may not be accompanied by the peer; monitoring of work towards the goals and review of the primary care visit; and an ending, to include a review and summary of the client's achievements, updating the well-being map and encouragement to continue to utilise it.

The network mapping process contributes to reflexivity, allowing participants to become more aware of the resources they have in networks to support physical health, and make positive behavioural change. Physical health can be activated indirectly through person-centred goals. A person might not be motivated to address physical health concerns, but they might be motivated to

For peer review only

address an area of well-being network map that matters to them. The peer can use these personal goals to indirectly improve the individual's physical health goals. For example, if they wish to join a book club to meet new people, the peer could suggest they walk/cycle to this club.

Development of the intervention

Working Group: A working group was set up of five people including: members of the research team; a representative from each of the other network mapping projects i.e. a community navigator from Hounslow and the PI of the Community Navigator Study; and a GP working in one of the boroughs we will recruit from. The working group met during the development phase of the study.

Key Informant Interviews: In each site key informant interviews were held with primary care mental health leads and peer support service managers to identify how the intervention would link with other provision and any clinical governance arrangements to be put in place. The PI liaised with mental health services through attendance at team meetings. The aim of this was to: introduce and generate a sense of ownership over the idea of the intervention; consult on the arrangements identified through the key informant interviews; discuss how to identify potential participants and agree the arrangements with any amendments needed.

Pre-pilot of the mapping process: The tool was adapted by the research team and peer support workers who helped to refine and streamline the process. It was then tested by peer support workers on each other and with the researcher. The research team observed and documented this process; data was field notes, interview transcripts and workshop outputs.

During the development of the intervention, two goals for the service user to work towards were added. The first goal was to visit a primary care service for a physical health appointment; the second was to create a behaviour change with a view to improving physical health. The aim was for the peer to encourage service users to utilise the map when working towards these goals as well as supporting them to attend an appointment by offering to accompany them.

Stages

The research will be conducted in three stages. Stage one comprises development of the intervention through key informant interviews and consulting a working group and producing a preliminary theory of change. Stage two comprises observing the training of peer support workers

For peer review only

and use of well-being network mapping; pilot mapping interviews; and piloting the intervention (n=24). Stage three will include stakeholder workshops and finalisation of the theory of change model.

Theory of change

The development of the intervention will be informed by a theory of change model (figure 1). This will help to identify the desired impacts and outcomes and the mechanism by which these are achieved. To refine the theory of change for further testing, we will use a mixed methods approach, collating quantitative outcome measures (pre and post intervention), and qualitative interviews and focus groups with both participants and PSWs. We will also conduct weekly supervision with PSWs and observe some of the intervention sessions.

<Insert figure 1 here>

Figure 1: Preliminary Theory of change model.

Sample size

The target sample size of 24 is based on the pragmatics of testing the feasibility of delivering this intervention to inform a larger scale study, therefore this sample size is appropriate (33).

Settings

The intervention will be conducted across two London boroughs.

Baseline and follow up procedures

Baseline data collection will take place following consent and before the initial meeting with the PSW. Participants will meet with a PSW and begin the intervention. After finishing the intervention, participants will be asked to complete outcome measures at four months from baseline, before completing a semi structured qualitative interview. PSWs will also take part in a semi structured qualitative interview and a focus group. The qualitative interviews will document the participant's view of their physical health and its management; experiences of the intervention; ways in which it worked; identify mechanisms through which the mapping helped lead people to take action on their physical health; and factors which limited its helpfulness; and how it could be improved. A focus group with all PSWs across both sites will seek feedback on use of the mapping tool;

For peer review only

discuss the actions planned versus those taken, and reasons for deviation; problems encountered and methods for solving them; and the outcomes achieved.

Participants will be compensated £10 for each of their baseline and follow up assessments and interviews. A further £15 will be given for focus group participation.

Outcomes

The peer support change model developed by Gillard at al (34) was used to identify outcomes and hence, the following outcomes will be measured pre intervention and at a 4 month follow up after the intervention has been completed:

Service use will be measured using an adapted version of the Client Service Receipt Inventory (35). Health and social care, informal care and use of leisure activities were measured. Primary care and secondary care (for physical health care) will be used as intermediate outcomes on the pathway to improved physical health; likewise leisure activities with potential benefit for physical health such as those involving exercise.

Social capital will be measured using the Resource Generator UK (36) (RG-UK). The RG-UK asks participants if they have or could obtain access to each of 27 skills or resources within their social network within one week. It then asks the nature of the social tie through which they could access each skill or resource. The instrument has four sub-scales: domestic resources, personal skills, expert advice and problem solving resources. It has good reliability and validity, and has been used in samples of people with mental health problems e.g., producing valid results. The RG-UK does not fully measure access to resources related to health, the primary interest of this study. Consequently an additional subscale for the RG-UK was used, developed by Pinfold et al (28), which captures health and well-being related resources.

Social network quality and quantity will be measured using the Luben Social Network Scale is a 6-item self-report measure assessing quantity and quality of contact with family and friends (37). Three questions to capture acquaintances were added to this measure.

Hope will be measured using the Herth Hope Index (38), a 12 item self-report measure designed to measure levels of hope in adults in clinical settings, increasingly used in mental health studies.

Mental health self-efficacy will be measured using the Mental Health Confidence Scale (39), an 11 item self-report measure based on theories of self-efficacy and research about self-help in mental health.

For peer review only

Mental well-being will be measured using the Short Warwick-Edinburgh Mental Well-being Scale (SWEMWBS) (40), a 7-item measure designed to measure mental well-being. The positively worded questions focus on assessing mental well-being as opposed to mental health difficulties.

Physical health will be measured using the EuroQol Five Dimensions Questionnaire (41), a two-part questionnaire assessing the objective and subjective health status of participants. The measure can be used to compute a Quality Adjusted Life Year (QALY) (42), an outcome frequently used in cost analyses.

Process evaluation

With the permission of both PSW and participant, the project researcher will observe intervention sessions. The aim of this observation is to evaluate the extent of the use of the mapping tool; whether and if so how it is used to inform goal setting and planning; and whether its use creates any difficulties in goal setting and planning. We will record information provided during supervision sessions between the research team and the PSWs.

Proposed data analysis

Quantitative data: Feasibility of the intervention will be assessed by rates of recruitment of peers and of clients at each site; implementation of the intervention – i.e. what proportions of participants had each of 0-6 sessions with a peer and what proportion visited their GP or other primary care service for a physical health issue. Feasibility of the evaluation will be assessed by completion rates of interviews and measures of peers and clients; rates of attendance at focus groups.

Qualitative data: Data from the first two participants at each site will be rapidly analysed by the study researchers with the main aim of identifying any feasible modifications needed to the intervention or study design. We will use a focussed thematic analysis approach (43) to identify: contextual barriers and facilitating factors to the successful implementation of the intervention with respect to the desired outcomes; the appropriateness of the selected outcomes (above); and mechanisms of action of the intervention. Multiple coding will be conducted on five transcripts to allow researchers to identify and discuss any alternative interpretations. The study researcher will input all coding using NVivo, with cross checks by another team member. We will hold analysis meetings to review transcripts and develop coding frames. Further synthesis meetings will be required once coding is complete.

For peer review only

Collated feedback

Stakeholders from both boroughs will be brought together to discuss the results. The outputs following the workshop will include: (1) agreed documentation for use including guides for both PSWs and clients to support the model, (2) select and refine outcomes for a randomised controlled trial, (3) manuals to document the well-being mapping process – one for clients and one for well-being partners.

Ethics and dissemination

Ethics approval for this study has been obtained from the Heath and Research Authority (HRA) and the London- Chelsea Regional Ethics Committee (REC); ref 17/LO/0585.

We plan to disseminate the results to the participants who received the intervention and the PWSs who delivered the intervention. We will also share the results with the NHS trusts we recruited from, the peer support services from which the PSWs were recruited, primary care mental health leads and commissioners. We plan to publish the findings in peer-reviewed journals and share our findings at academic conferences.

For peer review only

REFERENCE LIST

1. Wahlbeck K, Westman J, Nordentoft M, et al. Outcomes of Nordic mental health systems: life expectancy of patients with mental disorders. *British Journal of Psychiatry*. 2011.
2. Osborn DPJ, Nazareth I, King MB. Physical activity, dietary habits and Coronary Heart Disease risk factor knowledge amongst people with severe mental illness. *Soc Psychiatry Psychiatr Epidemiol*. 2007October01;42(10):787-93.
3. Matcham F, Carroll A, Chung N, et al. Smoking and common mental disorders in patients with chronic conditions: an analysis of data collected via a web-based screening system. *Gen Hosp Psychiatry*. 2017;45:12-8.
4. Reilly S, Planner C, Hann M, et al. The role of primary care in service provision for people with severe mental illness in the United Kingdom. *PloS one*. 2012;7(5):e36468.
5. Gillard S. The redistribution of mental health care: good, bad or just a bit ugly? *J Ment Health* (Abingdon, England). 2015 Dec;24(6):339-41.
6. Phelan M, Stradins L, Morrison S. Physical health of people with severe mental illness: can be improved if primary care and mental health professionals pay attention to it. *BMJ*. 2001;322(7284):443.
7. Bittles AH, Petterson B, Sullivan S, et al. The influence of intellectual disability on life expectancy. *The Journals of Gerontology Series A: Biological Sciences and Medical Sciences*. 20021July 2002;57(7):M470-M2.
8. Practitioners RCoG. A step by step guide for GP practices. 2010.

9. Corrigan PW, Mittal D, Reaves CM, et al. Mental health stigma and primary health care decisions. *Psychiatry Res*. 2014Aug15;218(1-2):35-8.
10. van Nieuwenhuizen A, Henderson C, Kassam A, et al. Emergency Department staff views and experiences on diagnostic overshadowing related to people with mental illness *Epidemiol Psychiatr Sci.* 2013;22(3):255-62.
11. Peckover S, Chidlaw RG. Too frightened to care? Accounts by district nurses working with clients who misuse substances. *Health Soc Care Community*. 2007 May;15(3):238-45.
12. Graber MA, Bergus G, Dawson JD, et al. Effect of a patient's psychiatric history on physicians' estimation of probability of disease. *J Gen Intern Med*. 2000 20000426DCOM-20000426;15:204-6.
13. Clarke DE, Dusome D, Hughes L. Emergency department from the mental health client's perspective. *Int J Ment Health Nurs*. 2007;16:126-31.
14. Jones S, Howard L, Thornicroft G. 'Diagnostic overshadowing': worse physical health care for people with mental illness. *Acta Psychiatr Scand*. 2008;118(3):169-71.
15. Lester H, Tritter JQ, Sorohan H. Patients' and health professionals' views on primary care for people with serious mental illness: focus group study. *BMJ*. 2005;330(7500):1122.
16. Bindman J, Johnson S, Wright S, et al. Integration between primary and secondary services in the care of the severely mentally ill: patients' and general practitioners' views. *BrJPsychiatry*. 1997;171:169-74.
17. Burns T, Greenwood, N., Kendrick, T., et al. Attitudes of general practitioners and community mental health team staff towards the locus of care for people with chronic psychotic disorders. *Int J Psychiatry Clin Pract*. 2000;6:67-71.
18. Lester H, Gask L. Delivering medical care for patients with serious mental illness or promoting a collaborative model of recovery? *BrJPsychiatry*. 2006;188:401-2.
19. Roberts L, Roalfe A, Wilson S, et al. Physical health care of patients with schizophrenia in primary care: a comparative study. *Fam Pract*. 2007;24(1):34-40.
20. Pitt V, Lowe D, Hill S, et al. Consumer-providers of care for adult clients of statutory mental health services. *Cochrane Database Syst Rev.* 2013;3:CD004807.
21. Galia SM, Zlatka R, Vasudha G, et al. Benefits and Mechanisms of Recovery Among Peer Providers With Psychiatric Illnesses. *Qual Health Res.* 20112012/03/01;22(3):304-19.
22. Moran GS, Russinova Z, Stepas K. Toward understanding the impact of occupational characteristics on the recovery and growth processes of peer providers. *Psychiatr Rehabil J*. 2012;35(5):376.
23. Kelly E, Duan L, Cohen H, et al. Integrating behavioral healthcare for individuals with serious mental illness: A randomized controlled trial of a peer health navigator intervention. *Schizophr Res.* 2016Oct25.
24. Druss BG, Zhao L, von Esenwein SA, et al. The Health and Recovery Peer (HARP) Program: a peer-led intervention to improve medical self-management for persons with serious mental illness. *Schizophr Res*. 2010May;118(1-3):264-70.
25. Goldberg RW, Dickerson F, Lucksted A, et al. Living well: an intervention to improve self-management of medical illness for individuals with serious mental illness. *Psychiatr Serv.* 2013Jan;64(1):51-7.
26. Sweet D, Byng R, Webber M, et al. Personal well-being networks, social capital and severe mental illness: exploratory study. *The British Journal of Psychiatry*. 2017Oct;211(6):117.
27. Connecting People study http://connectingpeoplestudy.net/.
28. Pinfold V, Sweet D, Porter I, et al. Improving community health networks for people with severe mental illness: a case study investigation. *Health Services and Delivery Research*. 2015 Feburary 2015;3(5).
29. Pinfold V, Sweet, D. Wellbeing networks and asset mapping. http://mcpin.org/wp-content/uploads/wellbeing-networks-SW-VERSION.pdf 2015.
30. Sweet D, Byng R, Webber M, et al. Personal well-being networks, social capital and severe mental illness: exploratory study. *The British Journal of Psychiatry*. 2017.

For peer review only

31. Kail A, Lumley, T. The theory of change – the beginning of making a difference. . London: New Philanthropy Capital; 2012.
32. Harris E, Hodgson, L., Noble, J. Creating your theory of change – NPC's practical guide. http://www.thinknpc.org/publications/creating-your-theory-of-change/: NPC; 2014.
33. Leon AC, Davis LL, Kraemer HC. The role and interpretation of pilot studies in clinical research. *J Psychiatr Res*. 2011;45(5):626-9.
34. Gillard S, Gibson SL, Holley J, et al. Developing a change model for peer worker interventions in mental health services: a qualitative research study. *Epidemiol Psychiatr Sci.* 2015Oct;24(5):435-45.
35. Beecham J, Knapp M. Costing psychiatric interventions. In: Thornicroft G, Brewin CR, Wing JK, editors. Measuring Mental Health Needs. London: Royal College of Psychiatrists, Gaskell; 1992.p.34-47.
36. Webber M, Corker E, Hamilton S, et al. Discrimination against people with severe mental illness and their access to social capital: findings from the Viewpoint survey. *EpidemiolPsychiatrSci*. 2013.
37. Lubben J, Blozik E, Gillmann G, et al. Performance of an abbreviated version of the Lubben Social Network Scale among three European community-dwelling older adult populations. *Gerontologist.* 2006Aug;46(4):503-13.
38. Herth K. Abbreviated instrument to measure hope: development and psychometric evaluation. J*ournal of advanced nursing*. 1992Oct;17(10):1251-9.
39. Carpinello SK, EL; Markowitz, FE; Pease, EA. The development of the Mental Health Confidence Scale: a measure of self-efficacy in individuals diagnosed with mental disorders. *Psychiatr Rehabil J.* 2000;23(3):236-43.
40. Tennant R, Hiller L, Fishwick R, et al. The Warwick-Edinburgh mental well-being scale (WEMWBS): development and UK validation. *Health Qual Life Out*. 2007Nov27;5.
41. Williams A. Euroqol - a New Facility for the Measurement of Health-Related Quality-of-Life. *Health policy*. 1990Dec;16(3):199-208.
42. Fanshel S, Bush JW. Health-Status Index and Its Application to Health-Services Outcomes. *Oper Res*. 1970;18(6):1021-66.
43. Braun VaC, V. Using thematic analysis in psychology. *Qual Res Psychol* 2006;3(2):77-101.

Data statement

The datasets used and/or analysed during the current study are available from the corresponding author on reasonable request.

Authors Statement

JC was the research assistant responsible for recruitment of participants to the study, data collection and peer supervision. JD was the research assistant responsible for helping develop the intervention, train the peer support workers and recruited participants to the study. CH was the chief investigator. DS and VP helped to develop the intervention. DS helped to train the peers. SG was the site lead and helped to train peers. JC drafted the paper and all authors provided edits and comments for its revision.

For peer review only

Acknowledgements

Bryn Lloyd-Evans, Amanda Perry, Sally Gomme (Hounslow), Nancy Kuchemann (GP and Southwark CCG clinical lead) and the Southwark and Sutton peer support services.

Funding

This research was supported by the Maudsley Charity and the National Institute for Health Research (NIHR) Collaboration for Leadership in Applied Health Research and Care South London (NIHR CLAHRC South London) at King's College Hospital NHS Foundation Trust. The views expressed in this article are of those of the authors and not necessarily those of the NHS, the NIHR, or the Department of Health and Social Care.

Conflicts of Interests

The authors declare that they have no competing interests

For peer review only

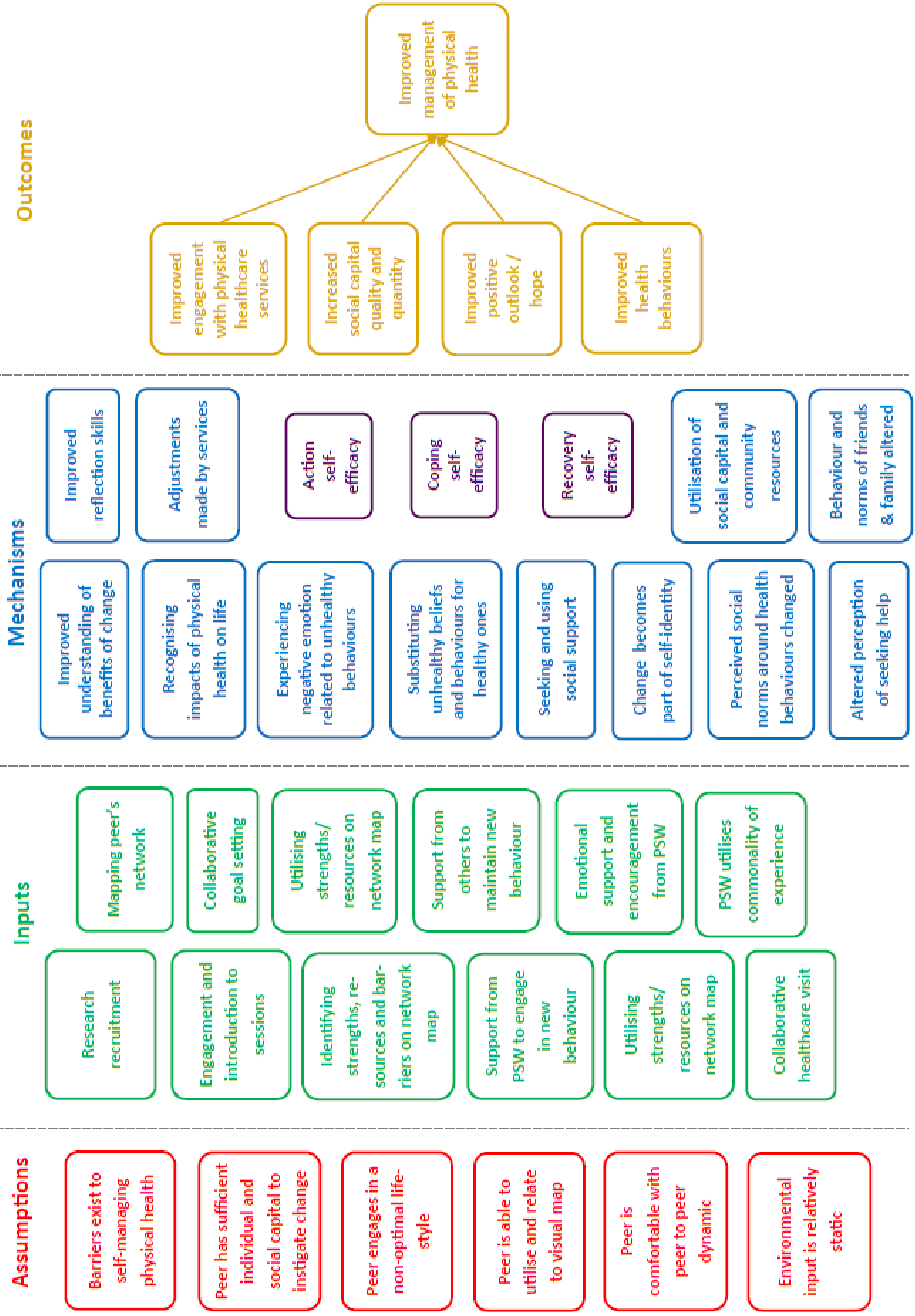
Outcomes
Improved management of physical health
Improved engagement with physical healthcare services
Increased social capital quality and quantity
Improved positive outlook / hope
Improved health behaviours
Mechanisms
Improved reflection skills
Adjustments made by services
Action self-efficacy
Coping self-efficacy
Recovery self-efficacy
Utilisation of social capital and community resources
Behaviour and norms of friends & family altered
Improved understanding of benefits of change
Recognising impacts of physical health on life
Experiencing negative emotion related to unhealthy behaviours
Substituting unhealthy beliefs and behaviours for healthy ones
Seeking and using social support
Change becomes part of self-identity
Perceived social norms around health behaviours changed
Altered perception of seeking help
Inputs
Mapping peer's network
Collaborative goal setting
Utilising strengths/ resources on network map
Support from others to maintain new behaviour
Emotional support and encouragement from PSW
PSW utilises commonality of experience
Research recruitment
Engagement and introduction to sessions
Identifying strengths, re-sources and bar-riers on network map
Support from PSW to engage in new behaviour
Utilising strengths/ resources on network map
Collaborative healthcare visit
Assumptions
Barriers exist to self-managing physical health
Peer has sufficient individual and social capital to instigate change
Peer engages in a non-optimal life-style
Peer is able to utilise and relate to visual map
Peer is comfortable with peer to peer dynamic
Environmental input is relatively static

BMJ Open

Managing transition from secondary mental to primary health care using peer-delivered well-being network mapping: protocol for a pilot study

Journal:	*BMJ Open*
Manuscript ID	bmjopen-2018-023768.R1
Article Type:	Protocol
Date Submitted by the Author:	23-Oct-2018
Complete List of Authors:	Collom, Jennifer; King's College London, Institute of Psychiatry, Psychology and Neuroscience<br>Davidson, Jonathan ; Institute of Psychiatry, Psychology and Neuroscience, Health Service and Population Research Department<br>Sweet, Daryl ; McPin Foundation<br>Gillard, Steve; St George's University of London, Division of Population Health Sciences and Education<br>Pinfold, Vanessa; McPin Foundation,<br>Henderson, Claire; King's College London, ; South London and Maudsley NHS Foundation Trust,
**Primary Subject Heading**:	Mental health
Secondary Subject Heading:	Mental health
Keywords:	Physical health, Peer support, Well-being network mapping, Intervention, Pilot, Protocol

SCHOLARONE™
Manuscripts

Managing transition from secondary mental to primary health care using peer-delivered well-being network mapping: protocol for a pilot study

Authors

Jennifer Collom[1] MSc

Jonathan Davidson[1] MA, MSc

Daryl Sweet[2] BSc Mres

Steve Gillard[3] PhD

Vanessa Pinfold[2] PhD

Claire Henderson[1] MRCPsych, PhD

For peer review only

Affiliations:

1. Health Service and Population Research Department, King's College London, Institute of Psychiatry, Psychology and Neuroscience, London, UK.
2. McPin Foundation, London, UK.
3. Population Health Research Institute, St Georges University of London, London, UK.

Correspondence to: Jennifer Collom, Health Service and Population Research Department, King's College London, Institute of Psychiatry, Psychology and Neuroscience, 16 De Crespigny Park, London, UK, SE5 8AF. Jennifer.Collom@kcl.ac.uk 0207 848 5087

Word Count: 4077

ABSTRACT

Introduction

Adults with Severe Mental Illness (SMI) have reduced life expectancy and many have comorbid physical health conditions. Primary care providers are experiencing increased demands to care for people with SMI. Barriers to accessing physical health care have been identified which negatively affect quality of care. We propose that Peer Support Workers (PSWs) could deliver an intervention to service users to promote their physical health by drawing on existing social support.
The objective of this research is to pilot a novel PSW led intervention, including Personal well-being network mapping, to improve access to primary care for physical health needs.

Methods and analysis

Twenty-four participants will be recruited from community based mental health teams in two boroughs of London. Each participant will be offered a 6 session intervention. Quantitative data will be collected pre and post intervention (at 4 month follow up). Qualitative interviews will be conducted with PSWs after completion of the intervention and with participants at a 4 month follow up. Some intervention sessions will be observed by a member of the research team. This is a pilot study with a small sample aiming to assess acceptability and feasibility of an intervention. We aim to use the results to refine the existing theory of change (ToC) and to optimise the intervention and its evaluation in a future randomised controlled trial (RCT). This study is strengthened by its potential clinical importance and origin in previous research where service users engaged with well-being network mapping.

For peer review only

Ethics and dissemination

This study has been approved by the London – Chelsea Regional Ethics Committee (ref: 17/LO/0585). The findings will be disseminated to participants, the NHS trusts that we recruited from, primary care mental health leads, commissioners and in peer-reviewed journals and academic conferences.

Keywords

Physical health, peer support, well-being network mapping, intervention, pilot, protocol.

ARTICLE SUMMARY

Strengths and limitations of this study

- The research will employ a mixed methods design; the qualitative data collected from participants and PSWs will aid the development of the intervention.
- The study will inform a larger scale research study into the intervention and in turn, could inform the development of a new approach to physical healthcare for people with SMI.
- Robust statistical analysis cannot be performed on the data due to the small sample size and no objective physical health measurements will be collected; this study does not aim to pilot collection of physical health data.
- The qualitative data collection employed by this study can be viewed as non-generalisable.

For peer review only

INTRODUCTION

People with severe mental illness (SMI) have greatly reduced life expectancy compared to the rest of the population, of 15-20 years depending on gender and diagnosis (1). High rates of smoking, obesity and low levels of exercise contribute to this (2, 3), as well as mental health related deaths (4). Engagement with primary care practitioners and facilities for health promotion (such as leisure facilities, smoking cessation groups, social activities) is increasingly important as many people with SMI have been discharged from community based mental health services, and care pathways have been redesigned to accelerate discharge after both referral and re-referral from primary care. Research published in 2012 found a high proportion of people with SMI are supported solely by a GP (31%) or with minimal secondary care input (5). This number is increasing as thresholds for admission to secondary mental health services are raised and more people discharged back to primary care (6).

However, this group has generally poor access to primary care for physical health needs. Primary care within current service configurations is not best placed to absorb increasing numbers of people with severe mental illness, discharged from secondary provision (5) and there is wide variation in the extent to which primary care is proactive in ensuring access to care for physical health problems (7).

Accommodations for those with SMI could be made within health care services, such as extended appointment times, to allow for discussion and treatment of both mental and physical health. Such accommodations are feasible within these setting and have been implemented for people with learning disabilities (8), another group who experience poorer physical health and lower life expectancies than the general population (8).

Attitudes to people with severe mental illnesses may create barriers to responses to physical health complaints. Research has outlined how many UK GPs: are less keen on supporting people with SMI than other medical conditions (9); lack specialist knowledge and skills (10); and have clear ideas that their role should be limited to physical health checks and providing medication (11, 12). In the USA, Corrigan and colleagues have found that primary care professionals working in the US Veteran’s Health

For peer review only

Administration. who endorsed stigmatizing characteristics of a patient with schizophrenia described in a vignette were more likely to believe the patient would not adhere to treatment; as a result, they were less likely to refer to a specialist or refill his prescription (13). There is also evidence from the US that family physicians are less likely to believe that patients with previous depression have serious medical conditions causing physical symptoms, leading to a greater reluctance to carry out investigations for such symptoms (14). This may reflect the misattribution of physical symptoms to pre-existing mental illness (15), a phenomenon known as 'diagnostic overshadowing' (16). A qualitative interview study of emergency department nurses and doctors (17) revealed that this is a fairly well recognised problem that can lead to adverse consequences varying from delay in treatment to death. This study also found that some participants avoid people who are experiencing symptoms of mental illness due to fear of violence, which may also adversely affect quality of care (17). Fear of patients with substance abuse problems has also been expressed by district nurses, with the consequent risk of suboptimal care (18).

Supported access and empowerment are thus needed for improved physical health care in this group. These functions may be carried out by professionals is specialist mental health care, however caseload sizes may not permit the time needed to focus on physical health and those whose mental health is fairly stable are likely to be discharged to primary care (19). Evidence from a number of trials reviewed recently suggests that some practitioner roles can be performed by trained and supervised Peer Support Workers (PSWs) leading to similar outcomes as when they are undertaken by mental health staff (20). PSWs are people with their own experience of mental health problems who use this experience to help others. They are becoming increasingly frequently employed within health services (21). There is also a potential longer term advantages of employing peer workers in this role; it creates a rung on the career ladder for the peer, which is also visible to the person in receipt of their support, encouraging recipients to in turn become a peer supporter or take on other similar roles (22).

For peer review only

Recent systematic reviews have evidenced the benefit of peer led interventions on physical health outcomes for people with SMI. Authors found studies measuring service use, self-related health, and quality of life amongst other outcomes (23, 24). They concluded that the most beneficial interventions focused on self-management of health behaviours and that peer led interventions had the most significant impact on hope and quality of life; these reviews support the outcomes measured in the current study and the need for well-developed peer led interventions.

This project draws on peer-led (25, 26) and peer co-facilitated (27) interventions in the US to improve self-management of physical health and increase uptake of primary care services for physical health conditions, and on UK research exploring social networks and their potential resources to support people with SMI. It is important to investigate peer support interventions for physical health in the UK, which has a different health system and different peer support services to the US. In the UK, the first well-being network mapping was developed by the McPin Foundation (28) following their study of social networks and how they relate to personal well-being (29). Several other studies (30-32) have drawn on this and other work on social networks to seek to address social isolation, by encouraging network development or enhancement using workers to empower and signpost individuals to opportunities locally to build connections to people, places and activities. Support networks are made up of social contacts, and recovery is linked to meaningful activities, identity and sense of purpose in life, empowerment, hope and connectedness. Further, community places and spaces constitute the environments in which people look after their health and well-being and build social networks. Well-being network mapping is asset based, strengths focused and through structured conversations may facilitate engagement and planning to address identified health and social care needs. The use of this tool will allow PSWs to assess the client's relationship with primary care, in the context of other health related behaviours and relationships which can promote or adversely affect health. The PSW will work with their client to utilise the connections established using the map with the aim of improving well-being in a sustained way, with a focus on access for physical health needs.

For peer review only

The project adopts a theory of change approach (33, 34). This is a method for developing a framework for delivering change, and explaining the mechanisms for doing so. It covers assumptions, inputs, and mechanisms and suggests appropriate outcomes based on underlying processes in order to better understand what is involved in achieving sustained long term change. Previous work on well-being network mapping has not employed this approach. It is hoped that the theory of change will improve current understanding of how people move from identifying resources and barriers in their networks, to making positive changes to their physical health and access to healthcare.

Aims and objectives

The aims of the project are:

1. To develop a localised model for a peer well-being network mapping intervention in two boroughs.
2. To produce a theory of change, mapping out assumptions, inputs, mechanisms and outcomes.
3. To deliver the intervention in two boroughs and assess impact on people with SMI and the PSWs.
4. To inform the development of a future feasibility trial to assess feasibility of a pragmatic trial of the effectiveness of the intervention with respect to access to primary care for physical health needs.

This paper describes work done for aims 1 and 2 and the protocol for aims 3 and 4.

METHODS AND ANALYSIS

Design

This study will pilot an intervention to improve physical healthcare utilising well-being network mapping delivered by PSWs. The intervention addresses barriers to accessing

For peer review only

physical healthcare and employs behavioural activation through goal setting for people with SMI. All participants will receive the intervention. The research will be conducted in three stages, the first of which is complete. Stage one comprises development of the intervention through key informant interviews and consulting a working group and producing a preliminary theory of change. Stage two comprises observing the training of PSWs and use of well-being network mapping; pilot mapping interviews; and piloting the intervention (n=24). Stage three will include a stakeholder workshop and finalisation of the theory of change model.

Settings

The intervention will be conducted across two London boroughs (local government areas). Participants will be recruited from community based mental health teams providing care for people with SMI. In one borough, the four teams we will recruit from specifically provide services for people with psychosis. In the other, the three teams work with people with psychosis and other conditions such as depression and anxiety.

Stage 1 Development of the intervention

Working Group: A working group was set up of five people including: members of the research team; a representative from each of the other network mapping projects i.e. a community navigator from Hounslow and the PI of the Community Navigator Study; and a GP working in one of the boroughs we will recruit from. The working group met during the development phase of the study.

Key Informant Interviews: In each site key informant interviews were held with primary care mental health leads and peer support service managers to identify how the intervention would link with other provision and any clinical governance arrangements to be put in place. The PI liaised with mental health services through attendance at team meetings. The aim of this was to: introduce and generate a sense of ownership over the idea of the intervention; consult on the arrangements identified through the key

For peer review only

informant interviews; discuss how to identify potential participants and agree the arrangements with any amendments needed.

Pre-pilot of the mapping process: A manual was drafted by the research team and PSWs who helped to refine and streamline the process. It was then tested by PSWs on each other and with the researcher. The research team observed and documented this process; data was field notes, interview transcripts and workshop outputs.

During the development of the intervention, two goals for the service user to work towards were added. The first goal was to visit a primary care service for a physical health appointment; the second was to create a behaviour change with a view to improving physical health. The aim was for the peer to encourage service users to utilise the map when working towards these goals as well as supporting them to attend an appointment by offering to accompany them.

Theory of change

The development of the intervention will be informed by a theory of change model (figure 1). This will help to identify the desired impacts and outcomes and the mechanism by which these are achieved. To refine the theory of change for further testing, we will use a mixed methods approach, collating quantitative outcome measures (pre and post intervention), and qualitative interviews and focus groups with both participants and PSWs. We will also conduct weekly supervision with PSWs and observe some of the intervention sessions.

<Insert figure 1 here>

Figure 1: Preliminary Theory of change model.

Stage 2 Piloting

For peer review only

Recruitment

A member of the research team will approach clinical staff at community based mental health team centres. They will be informed about the research, the inclusion/exclusion criteria, and given an information script. This will provide clinical staff with details of the study to relay to possible participants. Staff will be asked to consider their entire caseload, which usually consists of up to 30 people, and to contact eligible service users as agreed with the Research Ethics Committee. When clinical staff receive agreement from a service user, a meeting will be organised; during which, a researcher will provide a patient information sheet and answer any questions. Participants will be offered a period of time, usually at least 24 hours, to consider their involvement in the study according to IRAS guidance(35), before providing consent to participant in the study.

Sample size

The target sample size of 24 is based on the pragmatics of testing the feasibility of delivering this intervention to inform a larger scale study, therefore this sample size is appropriate (36).

Inclusion and exclusion criteria

The following inclusion criteria will be used: 1) approaching discharge from community based mental health services, as this group are soon to cease contact with mental health professionals who encourage healthy behaviours and help seeking for physical health; 2) at risk of deterioration in physical health due to poor access to primary care; 3) demonstrated failure to organise/attend primary care appointments for co-morbid physical health difficulties; 4) aged 18 or over.

People will not be eligible to take part in the study if they: 1) lack capacity to provide informed consent; this will be assessed during the consent process as the ability to

For peer review only

retain, weigh up and make decisions based on the information provided; 2) possess a high risk of harm to others, as judged by their clinical team; 3) are unable to communicate in English; 4) are currently an inpatient on a psychiatric hospital; and 5) nonattendance of discharge planning meeting with subsequent discharge.

Patient and public involvement

PSWs, who have lived experience of mental health problems, were involved in the development of the intervention through their provision of feedback on the draft manual, and will deliver and feed back their views the intervention. We also brought together a group of peer support workers and others involved in projects with the McPin Foundation that including a mapping component, as advisors. They helped refine the mapping process, contributed to the planned training programme and inputted into the draft ToC.

The intervention

Peer support workers: PSWs (n=6) were recruited from within existing peer support services in the two London boroughs (three per borough). The PSWs were paid to take on the peer well-being role. We aimed that each PSW will work with up to 4 clients. The training took place across two days. One day focused on the intervention, including the PSWs carrying out the mapping intervention with one another and development of the manual. The second day was a refresher on the key aspects of peer support. Training was delivered by a network mapping researcher (DS), a researcher with expertise on peer support (SG), and a researcher who lead on intervention development with input from the whole study team (JD). PSWs will be supported with use of the well-being mapping tool through regular supervision with the research team and their line manager.

Over six sessions, the intervention aims to cover the following topics: engagement of the client with the peer and the intervention; mapping of the client's network (see figure

For peer review only

2 for an example map), including people and services which contribute positively, negatively, or both, to the client's well-being; goal setting for physical health and planning; a visit to a primary care service e.g. their GP's surgery, dentist, or sexual health clinic, to which the client may or may not be accompanied by the peer; monitoring of work towards the goals and review of the primary care visit; and an ending, to include a review and summary of the client's achievements, updating the well-being map and encouragement to continue to utilise it. The visit to a primary care service will be prepared for collaboratively using resources which aim to prevent diagnostic overshadowing. For example, topics to be discussed in the appointment will be pre-agreed between the PSW and service user.

<Insert figure 2 here>

Figure 2: Example of a network map.

Some people with SMI may not have robust or wide social networks. In these cases, the PSWs will make suggestions for ways to expand the person's social network. The observations, notes from supervision with PSWs, and qualitative feedback will identify whether additions to the intervention are needed to try to build social networks.

The network mapping process contributes to reflexivity, allowing participants to become more aware of the resources they have in networks to support physical health, and make positive behavioural change. Physical health can be activated indirectly through person-centred goals. A person might not be motivated to address physical health concerns, but they might be motivated to address an area of well-being network map that matters to them. The peer can use these personal goals to indirectly improve the individual's physical health goals. For example, if they wish to join a book club to meet new people, the peer could suggest they walk/cycle to this club.

Participants can arrange to meet with their PSW in a location that suits them both. This may include private rooms at their community based mental health service, a library or a café. The six sessions will in most cases take place across 6 weeks, however this time period can be elongated to reflect the needs of the service user and to allow a primary

For peer review only

care visit. Each session will last up to two hours. The sessions are guided by the manual, however the map and the goals created will be personalised to the individual.

Baseline and follow up procedures

Baseline data collection will take place following consent and before the initial meeting with the PSW. Participants will meet with a PSW and begin the intervention. After finishing the intervention, participants will be asked to complete outcome measures at four months from baseline, before completing a semi structured qualitative interview. PSWs will also take part in a semi structured qualitative interview and a focus group. The qualitative interviews will document the participant's view of their physical health and its management; experiences of the intervention; ways in which it worked; identify mechanisms through which the mapping helped lead people to take action on their physical health; and factors which limited its helpfulness; and how it could be improved. A focus group with all PSWs across both sites will seek feedback on use of the mapping tool; discuss the actions planned versus those taken, and reasons for deviation; problems encountered and methods for solving them; and the outcomes achieved.

Participants will be compensated £10 for each of their baseline and follow up assessments and interviews. A further £15 will be given for focus group participation.

Outcomes

The peer support change model developed by Gillard at al (37) was used to identify outcomes and hence, the following outcomes will be measured pre intervention and at a 4 month follow up after the intervention has been completed:

Service use will be measured using an adapted version of the Client Service Receipt Inventory (38). Health and social care, informal care and use of leisure activities were measured. Primary care and secondary care (for physical health care) will be used as

For peer review only

intermediate outcomes on the pathway to improved physical health; likewise leisure activities with potential benefit for physical health such as those involving exercise.

Social capital will be measured using the Resource Generator UK (39) (RG-UK). The RG-UK asks participants if they have or could obtain access to each of 27 skills or resources within their social network within one week. It then asks the nature of the social tie through which they could access each skill or resource. The instrument has four sub-scales: domestic resources, personal skills, expert advice and problem solving resources. This measure assesses service use and healthy behaviours to capture improvements to management of physical health. It reflects reciprocal relationships, so change may be due to either the participant, members of their network, or both. It has good reliability and validity, and has been used in samples of people with mental health problems e.g., producing valid results. The RG-UK does not fully measure access to resources related to health, the primary interest of this study. Consequently an additional subscale for the RG-UK was used, developed by Pinfold et al (40), which captures health and well-being related resources.

Social network quality and quantity will be measured using the Luben Social Network Scale is a 6-item self-report measure assessing quantity and quality of contact with family and friends (41). Three questions to capture acquaintances were added to this measure.

Hope will be measured using the Herth Hope Index (42), a 12 item self-report measure designed to measure levels of hope in adults in clinical settings, increasingly used in mental health studies. This measure has been found to hold divergent and construct validity(42).

Mental health self-efficacy will be measured using the Mental Health Confidence Scale (43), an 11 item self-report measure based on theories of self-efficacy and self-help. The scale was shown to have good internal consistency and clinical utility (44).

Mental well-being will be measured using the Short Warwick-Edinburgh Mental Well-being Scale (SWEMWBS) (45), a 7-item measure designed to measure mental well-being, found to have good reliability and validity for people with SMI (46). The positively

For peer review only

worded questions focus on assessing mental well-being as opposed to mental health difficulties.

Physical health will be measured using the EuroQol Five Dimensions Questionnaire (47), a two-part questionnaire assessing the objective and subjective health status of participants. The measure can be used to compute a Quality Adjusted Life Year (QALY) (48), an outcome frequently used in cost analyses, and has been found to be a valid measure among people with schizophrenia (49).

Some outcomes and mechanisms will not be measured and are shown as underlined in the preliminary ToC model (see figure 1). These are intermediate outcomes and we have prioritised outcomes further along the ToC model, to reduce respondent burden.

For peer review only

Process evaluation

With the permission of both PSW and participant, the project researcher will observe PSWs delivering some sessions of the intervention to participants in vivo. The aim of this observation is to evaluate the extent of the use of the mapping tool; whether and if so how it is used to inform goal setting and planning; and whether its use creates any difficulties in goal setting and planning. We will record information provided during supervision sessions between the research team and the PSWs.

Proposed data analysis

Quantitative data: Feasibility of the intervention will be assessed by rates of recruitment of peers and of clients at each site; implementation of the intervention – i.e. what proportions of participants had each of 0-6 sessions with a peer and what proportion visited their GP or other primary care service for a physical health issue. Feasibility of the evaluation will be assessed by completion rates of interviews and measures of peers and clients; rates of attendance at focus groups. Floor to ceiling effects of measures will be identified, as well as the level of outcome measure completion.

Qualitative data: Data from the first two participants at each site will be rapidly analysed by the study researchers with the main aim of identifying any feasible modifications needed to the intervention or study design. We will use a focussed thematic analysis approach (50) to identify: contextual barriers and facilitating factors to the successful implementation of the intervention with respect to the desired outcomes; the appropriateness of the selected outcomes (above); and mechanisms of action of the intervention. Multiple coding will be conducted on five transcripts to allow researchers to identify and discuss any alternative interpretations. The study researcher will input all coding using NVivo, with cross checks by another team member. We will hold analysis meetings to review transcripts and develop coding frames. Further synthesis meetings will be required once coding is complete.

Stage 3. Collated feedback

Stakeholders from both boroughs will be brought together to discuss the results. The outputs following the workshop will include: 1) agreed documentation for use including guides for both PSWs and clients to support the model; 2) select and refine outcomes for a randomised controlled trial; 3) manuals to document the well-being mapping process – one for clients and one for well-being partners.

Ethics and dissemination

Ethics approval for this study has been obtained from the Heath and Research Authority (HRA) and the London- Chelsea Regional Ethics Committee (REC); ref 17/LO/0585.

We plan to disseminate the results to the participants who received the intervention and the PSWs who delivered the intervention. We will also share the results with the NHS trusts we recruited from, the peer support services from which the PSWs were recruited, primary care mental health leads and commissioners. We plan to publish the findings in peer-reviewed journals and share our findings at academic conferences.

For peer review only

REFERENCE LIST

1. Wahlbeck K, Westman J, Nordentoft M, Gissler M, Laursen TM. Outcomes of Nordic mental health systems: life expectancy of patients with mental disorders. British Journal of Psychiatry. 2011.

2. Osborn DPJ, Nazareth I, King MB. Physical activity, dietary habits and Coronary Heart Disease risk factor knowledge amongst people with severe mental illness. Social Psychiatry and Psychiatric Epidemiology. 2007;42(10):787-93.

3. Matcham F, Carroll A, Chung N, Crawford V, Galloway J, Hames A, et al. Smoking and common mental disorders in patients with chronic conditions: an analysis of data collected via a web-based screening system. General hospital psychiatry. 2017;45:12-8.

4. John A, McGregor J, Jones I, Lee SC, Walters JT, Owen MJ, et al. Premature mortality among people with severe mental illness—New evidence from linked primary care data. 2018.

5. Reilly S, Planner C, Hann M, Reeves D, Nazareth I, Lester H. The role of primary care in service provision for people with severe mental illness in the United Kingdom. PLoS One. 2012;7(5):e36468.

6. Gillard S. The redistribution of mental health care: good, bad or just a bit ugly? J Ment Health. 2015;24(6):339-41.

7. Phelan M, Stradins L, Morrison S. Physical health of people with severe mental illness: can be improved if primary care and mental health professionals pay attention to it. BMJ: British Medical Journal. 2001;322(7284):443.

8. Bittles AH, Bev A. Petterson, Sheena G. Sullivan, Rafat Hussain, Emma J. Glasson, and Philip D. Montgomery. The influence of intellectual disability on life expectancy. The Journals of Gerontology Series A: Biological Sciences and Medical Sciences. 2002;57(7):M470 - M2.

9. Lester H, Tritter JQ, Sorohan H. Patients' and health professionals' views on primary care for people with serious mental illness: focus group study. BMJ. 2005;330(7500):1122.

For peer review only

10. Bindman J, Johnson S, Wright S, Szmukler G, Bebbington P, Kuipers E, et al. Integration between primary and secondary services in the care of the severely mentally ill: patients' and general practitioners' views. BrJPsychiatry. 1997;171:169-74.

11. Burns T, Greenwood, N., Kendrick, T., Garland, C. Attitudes of general practitioners and community mental health team staff towards the locus of care for people with chronic psychotic disorders. Int J Psychiatry Clin Pract. 2000;6:67-71.

12. Lester H, Gask L. Delivering medical care for patients with serious mental illness or promoting a collaborative model of recovery? BrJPsychiatry. 2006;188:401-2.

13. Corrigan PW, Mittal D, Reaves CM, Haynes TF, Han X, Morris S, et al. Mental health stigma and primary health care decisions. Psychiatry Res. 2014;218(1-2):35-8.

14. Graber MA, Bergus G, Dawson JD, Wood GB, Levy BT, Levin I. Effect of a patient's psychiatric history on physicians' estimation of probability of disease. Journal of General Internal Medicine. 2000;15:204-6.

15. Clarke DE, Dusome D, Hughes L. Emergency department from the mental health client's perspective. International Journal of Mental Health Nursing. 2007;16:126-31.

16. Jones S, Howard L, Thornicroft G. 'Diagnostic overshadowing': worse physical health care for people with mental illness. Acta Psychiatrica Scandinavica. 2008;118(3):169-71.

17. van Nieuwenhuizen A, Henderson C, Kassam A, Graham T, Murray J, Howard LM, et al. Emergency Department staff views and experiences on diagnostic overshadowing related to people with mental illness Epidemiology and Psychiatric Sciences. 2013;22(3):255-62.

18. Peckover S, Chidlaw RG. Too frightened to care? Accounts by district nurses working with clients who misuse substances. Health Soc Care Community. 2007;15(3):238-45.

19. Roberts L, Roalfe A, Wilson S, Lester H. Physical health care of patients with schizophrenia in primary care: a comparative study. Family Practice. 2007;24(1):34-40.

20. Pitt V, Lowe D, Hill S, Prictor M, Hetrick SE, Ryan R, et al. Consumer-providers of care for adult clients of statutory mental health services. Cochrane Database Syst Rev. 2013;3:CD004807.

For peer review only

21. Gillard S, Holley J, Gibson S, Larsen J, Lucock M, Oborn E, et al. Introducing New Peer Worker Roles into Mental Health Services in England: Comparative Case Study Research Across a Range of Organisational Contexts. 2015;42(6):682-94.

22. Moran GS, Russinova Z, Gidugu V, Yim JY, Sprague CJQHR. Benefits and mechanisms of recovery among peer providers with psychiatric illnesses. 2012;22(3):304-19.

23. Bellamy C, Schmutte T, Davidson L. An update on the growing evidence base for peer support. 2017;21(3):161-7.

24. Cabassa LJ, Camacho D, Vélez-Grau CM, Stefancic AJJopr. Peer-based health interventions for people with serious mental illness: a systematic literature review. 2017;84:80-9.

25. Kelly E, Duan L, Cohen H, Kiger H, Pancake L, Brekke J. Integrating behavioral healthcare for individuals with serious mental illness: A randomized controlled trial of a peer health navigator intervention. Schizophr Res. 2016.

26. Druss BG, Zhao L, von Esenwein SA, Bona JR, Fricks L, Jenkins-Tucker S, et al. The Health and Recovery Peer (HARP) Program: a peer-led intervention to improve medical self-management for persons with serious mental illness. Schizophr Res. 2010;118(1-3):264-70.

27. Goldberg RW, Dickerson F, Lucksted A, Brown CH, Weber E, Tenhula WN, et al. Living well: an intervention to improve self-management of medical illness for individuals with serious mental illness. Psychiatr Serv. 2013;64(1):51-7.

28. Pinfold V, Sweet, D. Wellbeing networks and asset mapping. http://mcpin.org/wp-content/uploads/wellbeing-networks-SW-VERSION.pdf 2015 [

29. Sweet D, Byng R, Webber M, Enki DG, Porter I, Larsen J, et al. Personal well-being networks, social capital and severe mental illness: exploratory study. The British Journal of Psychiatry. 2017.

30. Lloyd-Evans B, Bone JK, Pinfold V, Lewis G, Billings J, Frerichs J, et al. The Community Navigator Study: a feasibility randomised controlled trial of an intervention to increase community connections and reduce loneliness for people with complex anxiety or depression. 2017;18(1):493.

For peer review only

31. Webber M, Reidy H, Ansari D, Stevens M, Morris DJRoSWP. Developing and modeling complex social interventions: Introducing the connecting people intervention. 2016;26(1):14-9.

32. Foundation TM. Wellbeing Networks and Mental Health: The McPin Foundation; 2018 [Available from: http://mcpin.org/networks-and-mental-health/.

33. Kail A, Lumley, T. The theory of change – the beginning of making a difference. . London: New Philanthropy Capital; 2012.

34. Harris E, Hodgson, L., Noble, J. Creating your theory of change – NPC's practical guide. http://www.thinknpc.org/publications/creating-your-theory-of-change/: NPC; 2014 [

35. (IRAS) IRAS. Collated Question-specific guidance for NHS REC Form. 2018 2018.

36. Leon AC, Davis LL, Kraemer HC. The role and interpretation of pilot studies in clinical research. Journal of psychiatric research. 2011;45(5):626-9.

37. Gillard S, Gibson SL, Holley J, Lucock M. Developing a change model for peer worker interventions in mental health services: a qualitative research study. Epidemiol Psychiatr Sci. 2015;24(5):435-45.

38. Beecham J, Knapp M. Costing psychiatric interventions. In: Thornicroft G, Brewin CR, Wing JK, editors. Measuring Mental Health Needs. London: Royal College of Psychiatrists, Gaskell; 1992. p. 34-47.

39. Webber M, Corker E, Hamilton S, Weeks C, Pinfold V, Rose D, et al. Discrimination against people with severe mental illness and their access to social capital: findings from the Viewpoint survey. EpidemiolPsychiatrSci. 2013.

40. Vanessa Pinfold DS, Ian Porter, Cath Quinn, Richard Byng, Chris Griffiths, Julie Billsborough, Doyo Gragn Enki, Ruth Chandler, Martin Webber, John Larsen, John Carpenter, and Peter Huxley. Improving community health networks for people with severe mental illness: a case study investigation. Health Services and Delivery Research. 2015;3(5).

41. Lubben J, Blozik E, Gillmann G, Iliffe S, von Renteln Kruse W, Beck JC, et al. Performance of an abbreviated version of the Lubben Social Network Scale among

For peer review only

three European community-dwelling older adult populations. Gerontologist. 2006;46(4):503-13.
42. Herth K. Abbreviated instrument to measure hope: development and psychometric evaluation. J Adv Nurs. 1992;17(10):1251-9.
43. Carpinello SK, EL; Markowitz, FE; Pease, EA. The development of the Mental Health Confidence Scale: a measure of self-efficacy in individuals diagnosed with mental disorders. Psychiatric Rehabilitation Journal. 2000;23(3):236-43.
44. Castelein S, van der Gaag M, Bruggeman R, van Busschbach JT, Wiersma DJPS. Measuring empowerment among people with psychotic disorders: a comparison of three instruments. 2008;59(11):1338-42.
45. Tennant R, Hiller L, Fishwick R, Platt S, Joseph S, Weich S, et al. The Warwick-Edinburgh mental well-being scale (WEMWBS): development and UK validation. Health and Quality of Life Outcomes. 2007;5.
46. Vaingankar JA, Abdin E, Chong SA, Sambasivam R, Seow E, Jeyagurunathan A, et al. Psychometric properties of the short Warwick Edinburgh mental well-being scale (SWEMWBS) in service users with schizophrenia, depression and anxiety spectrum disorders. 2017;15(1):153.
47. Williams A. Euroqol - a New Facility for the Measurement of Health-Related Quality-of-Life. Health Policy. 1990;16(3):199-208.
48. Fanshel S, Bush JW. Health-Status Index and Its Application to Health-Services Outcomes. Operations Research. 1970;18(6):1021-66.
49. Mulhern B, Mukuria C, Barkham M, Knapp M, Byford S, Brazier JJTBJoP. Using generic preference-based measures in mental health: psychometric validity of the EQ-5D and SF-6D. 2014;205(3):236-43.
50. Braun VaC, V. Using thematic analysis in psychology. Qualitative Research in Psychology. 2006;3(2):77 - 101.

Data statement

The datasets used and/or analysed during the current study are available from the corresponding author on reasonable request.

Contributorship Statement

For peer review only

JC was the research assistant responsible for recruitment of participants to the study, data collection and peer supervision. JD was the research assistant responsible for helping develop the intervention, train the peer support workers and recruited participants to the study. CH was the chief investigator. DS and VP helped to develop the intervention. DS helped to train the peers. SG was the site lead and helped to train peers. JC drafted the paper and all authors provided edits and comments for its revision.

Acknowledgements

Bryn Lloyd-Evans, Amanda Perry, Sally Gomme (Hounslow), Nancy Kuchemann (GP and Southwark CCG clinical lead) and the Southwark and Sutton peer support services.

Funding

This research was supported by the Maudsley Charity and the National Institute for Health Research (NIHR) Collaboration for Leadership in Applied Health Research and Care South London (NIHR CLAHR South London) at King's College Hospital NHS Foundation Trust. The views expressed in this article are of those of the authors and not necessarily those of the NHS, the NIHR or the Department of Health and Social Care.

Conflicts of Interest

The authors declare that they have no competing interests.

For peer review only

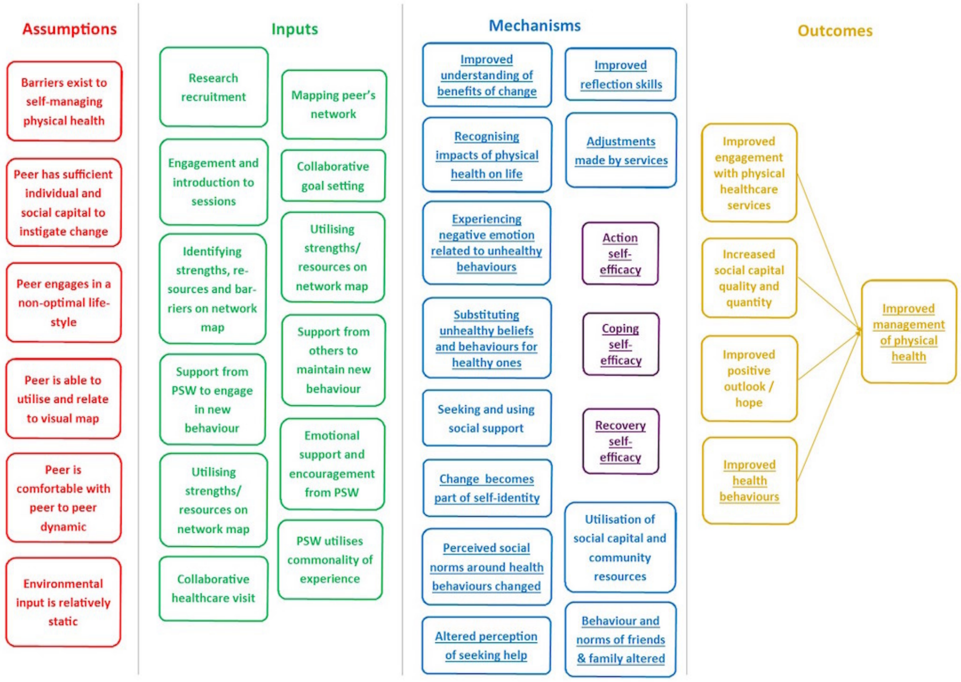

Figure 1: Preliminary Theory of change model.

90x63mm (300 x 300 DPI)

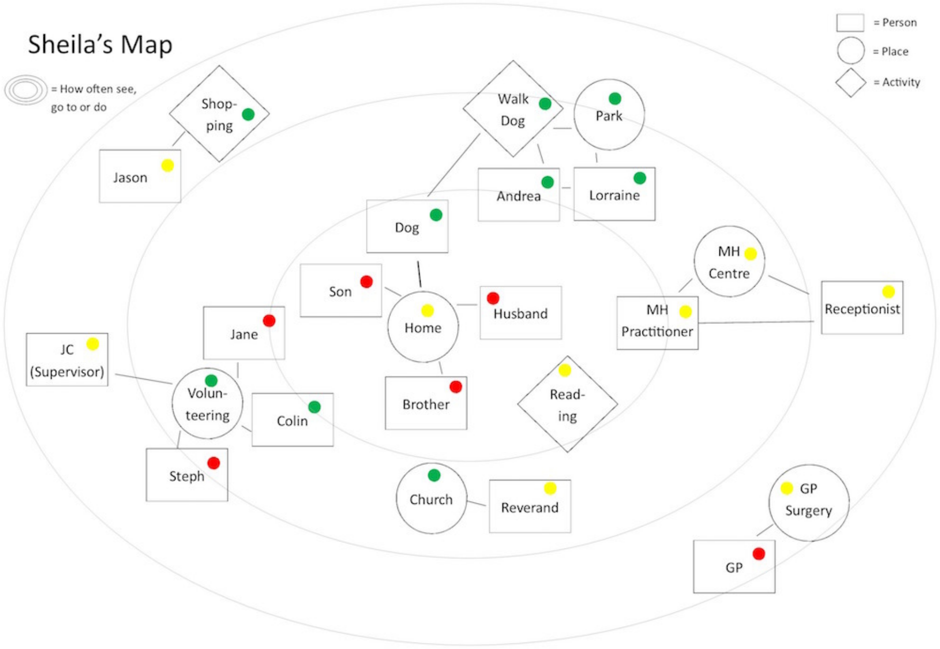

Figure 2: Example of a network map.

90x63mm (300 x 300 DPI)

BMJ Open

Development of a peer-led, network-mapping intervention to improve the health of individuals with severe mental illnesses: protocol for a pilot study

Journal:	*BMJ Open*
Manuscript ID	bmjopen-2018-023768.R2
Article Type:	Protocol
Date Submitted by the Author:	31-Jan-2019
Complete List of Authors:	Collom, Jennifer; King's College London, Institute of Psychiatry, Psychology and Neuroscience<br>Davidson, Jonathan ; Institute of Psychiatry, Psychology and Neuroscience, Health Service and Population Research Department<br>Sweet, Daryl ; McPin Foundation<br>Gillard, Steve; St George's University of London, Division of Population Health Sciences and Education<br>Pinfold, Vanessa; McPin Foundation,<br>Henderson, Claire; King's College London, ; South London and Maudsley NHS Foundation Trust,
**Primary Subject Heading**:	Mental health
Secondary Subject Heading:	Mental health
Keywords:	Physical health, Peer support, Well-being network mapping, Intervention, Pilot, Protocol

SCHOLARONE™
Manuscripts

Development of a peer-led, network-mapping intervention to improve the health of individuals with severe mental illnesses: protocol for a pilot study

Authors

Jennifer Collom[1] MSc

Jonathan Davidson[1] MA, MSc

Daryl Sweet[2] BSc Mres

Steve Gillard[3] PhD

Vanessa Pinfold[2] PhD

Claire Henderson[1] MRCPsych, PhD

For peer review only

Affiliations:

1. Health Service and Population Research Department, King's College London, Institute of Psychiatry, Psychology and Neuroscience, London, UK.
2. McPin Foundation, London, UK.
3. Population Health Research Institute, St Georges University of London, London, UK.

Correspondence to: Jennifer Collom, Health Service and Population Research Department, King's College London, Institute of Psychiatry, Psychology and Neuroscience, 16 De Crespigny Park, London, UK, SE5 8AF. Jennifer.Collom@kcl.ac.uk 0207 848 5087

Word Count: 4122

For peer review only

ABSTRACT

Introduction

Adults with Severe Mental Illness (SMI) have reduced life expectancy and many have comorbid physical health conditions. Primary care providers are experiencing increased demands to care for people with SMI. Barriers to accessing physical health care have been identified which negatively affect quality of care. We propose that Peer Support Workers (PSWs) could deliver an intervention to service users to promote their physical health by drawing on existing social support.

The aim of this research is to pilot a novel PSW led intervention, including Personal well-being network mapping, to improve access to primary care for physical health needs.

Methods and analysis

Twenty-four participants will be recruited from community-based mental health teams in two boroughs of London. Each participant will be offered a 6-session intervention. Quantitative data will be collected pre and post intervention (at 4-month follow up). Qualitative interviews will be conducted with PSWs after completion of the intervention and with participants at a 4 month follow up. Some intervention sessions will be observed by a member of the research team. This is a pilot study with a small sample aiming to assess acceptability and feasibility of an intervention. We aim to use the results to refine the existing theory of change (ToC) and to optimise the intervention and its evaluation in a future randomised controlled trial (RCT). This study is strengthened by its potential clinical importance and origin in previous research where service users engaged with well-being network mapping.

Ethics and dissemination

This study has been approved by the London – Chelsea Regional Ethics Committee (ref: 17/LO/0585). The findings will be disseminated to participants, the NHS trusts that we recruited from, primary care mental health leads, commissioners and in peer-reviewed journals and academic conferences.

Keywords
Physical health, peer support, well-being network mapping, intervention, pilot, protocol.

ARTICLE SUMMARY

Strengths and limitations of this study

- The research will employ a mixed methods design; the qualitative data collected from participants and PSWs will aid the development of the intervention.
- The study will inform a larger scale research study into the intervention and in turn, could inform the development of a new approach to physical healthcare for people with SMI.
- Robust statistical analysis cannot be performed on the data due to the small sample size and no objective physical health measurements will be collected; this study does not aim to pilot collection of physical health data.
- The qualitative data collection employed by this study can be viewed as non-generalizable.

For peer review only

INTRODUCTION

Background

People with severe mental illness (SMI) have greatly reduced life expectancy compared to the rest of the population, of 15-20 years depending on gender and diagnosis (1). High rates of smoking, obesity and low levels of exercise contribute to this (2, 3), as well as mental health related deaths (4). Engagement with primary care practitioners and facilities for health promotion (such as leisure facilities, smoking cessation groups, social activities) is increasingly important as many people with SMI have been discharged from community-based mental health services, and care pathways have been redesigned to accelerate discharge after both referral and re-referral from primary care. Research published in 2012 found a high proportion of people with SMI are supported solely by a primary care physician (called a General practitioner or GP in the UK) (31%), or with minimal specialist mental health care input (5). This number is increasing as thresholds for admission to specialist mental health services are raised and more people discharged back to primary care (6).

However, this group has generally poor access to primary care for physical health needs. Primary care within current service configurations is not best placed to absorb increasing numbers of people with severe mental illness discharged from secondary provision (5), and there is wide variation in the extent to which primary care is proactive in ensuring access to care for physical health problems (7).

Accommodations for those with SMI could be made within health care services, such as extended appointment times, to allow for discussion and treatment of both mental and physical health. Such accommodations are feasible within these settings and have been implemented for people with learning disabilities (8), another group who experience poorer physical health and a lower life expectancy than the general population (8).

Attitudes to people with severe mental illnesses may create a barrier to responding to physical health complaints. Research has outlined how many UK GPs: are less keen on supporting people with SMI than other medical conditions (9); lack specialist knowledge and skills (10); and have clear ideas that their role should be limited to physical health checks and providing medication (11, 12). In the USA, Corrigan and

colleagues found that primary care professionals working in the US Veteran's Health Administration, who endorsed stigmatizing characteristics of a patient with schizophrenia described in a vignette, were more likely to believe the patient would not adhere to treatment; as a result, they were less likely to refer to a specialist or refill their prescription (13). There is also evidence from the US that family physicians are less likely to believe that patients with previous depression have serious medical conditions causing physical symptoms, leading to a greater reluctance to carry out investigations for such symptoms (14). This may reflect the misattribution of physical symptoms to pre-existing mental illness (15), a phenomenon known as 'diagnostic overshadowing' (16). A qualitative interview study of emergency department nurses and doctors (17) revealed that this is a fairly well recognised problem that can lead to adverse consequences varying from delay in treatment to death. This study also found that some participants avoid people who are experiencing symptoms of mental illness due to fear of violence, which may also adversely affect quality of care (17). Fear of patients with substance abuse problems has also been expressed by district nurses who work in primary care, with the consequent risk of suboptimal care (18).

Peer support

Supported access and empowerment are thus needed for improved physical health care in this group. These functions may be carried out by professionals in specialist mental health care, however caseload size may not permit the time needed to focus on physical health and those whose mental health is fairly stable are likely to be discharged to primary care (19). Evidence from a number of trials reviewed recently suggests that some practitioner roles can be performed by trained and supervised Peer Support Workers (PSWs), leading to similar outcomes as when they are undertaken by mental health staff (20). PSWs are people with their own experience of mental health problems who use this experience to help others. They are becoming increasingly frequently employed within health services (21). There are also potential longer term advantages of employing peer workers in this role; it creates a rung on the career ladder for the peer, which is also visible to the person in receipt of their support, encouraging recipients to in turn become a peer supporter or take on other similar roles (22).

For peer review only

Recent systematic reviews have evidenced the benefit of peer-led interventions on physical health outcomes for people with SMI. Authors found studies measuring service use, self-related health, and quality of life amongst other outcomes (23, 24). They concluded that the most beneficial interventions focused on self-management of health behaviours and that peer-led interventions had the most significant impact on hope and quality of life; these reviews support the outcomes measured in the current study and the need for well-developed peer-led interventions.

This project draws on peer-led (25, 26) and peer co-facilitated (27) interventions in the US to improve self-management of physical health and increase uptake of primary care services for physical health conditions, and on UK research exploring social networks and their potential resources to support people with SMI. It is important to investigate peer support interventions for physical health in the UK, which has a different health system and different peer support services to the US. In the UK, the first well-being network mapping was developed by the McPin Foundation (28) following their study of social networks and how they relate to personal well-being (29). Several other studies (30-32) have drawn on this and other work on social networks to seek to address social isolation, by encouraging network development or enhancement using workers to empower and signpost individuals to opportunities locally to build connections to people, places and activities. Support networks are made up of social contacts, and recovery is linked to meaningful activities, identity and sense of purpose in life, empowerment, hope and connectedness. Further, community places and spaces constitute the environments in which people look after their health and well-being and build social networks. Well-being network mapping is asset based, strengths focused and through structured conversations may facilitate engagement and planning to address identified health and social care needs. The use of this tool will allow PSWs to assess the client's relationship with primary care, in the context of other health related behaviours and relationships, which can promote or adversely affect health. The PSW will work with their client to utilise the connections established using the map with the aim of improving well-being in a sustained way, and a focus on access for physical health needs.

The project adopts a theory of change approach (33, 34). This is a method for developing a framework for delivering change, and explaining the mechanisms for

For peer review only - http://bmjopen.bmj.com/site/about/guidelines.xhtml

doing so. It covers assumptions, inputs, and mechanisms and suggests appropriate outcomes based on underlying processes in order to better understand what is involved in achieving sustained long-term change. Previous work on well-being network mapping has not employed this approach. It is hoped that the theory of change will improve current understanding of how people move from identifying resources and barriers in their networks, to making positive changes to their physical health and access to healthcare.

Aims

The aims of the project are:

1. To develop a localised model for a peer well-being network mapping intervention in two boroughs.
2. To produce a theory of change, mapping out assumptions, inputs, mechanisms and outcomes.
3. To deliver the intervention in two boroughs and assess impact on people with SMI and the PSWs.
4. To inform the development of a future feasibility trial to assess feasibility of a pragmatic trial of the effectiveness of the intervention with respect to access to primary care for physical health needs.

This paper describes work done for aims 1 and 2 and the protocol for aims 3 and 4.

METHODS AND ANALYSIS

Design

This study will pilot an intervention to improve physical healthcare utilising well-being network mapping delivered by PSWs. The intervention addresses barriers to accessing physical healthcare and employs behavioural activation through goal setting for people with SMI. All participants will receive the intervention. The research will be conducted in three stages, the first of which is complete. Stage one comprises development of the intervention through key informant interviews and consulting a working group and producing a preliminary theory of change. Stage two comprises

For peer review only

observing the training of PSWs and use of well-being network mapping; pilot mapping interviews; and piloting the intervention (n=24). Stage three will include a stakeholder workshop and finalisation of the theory of change model.

Settings

The intervention will be conducted across two London boroughs (local government areas). Participants will be recruited from community-based mental health teams providing care for people with SMI. In one borough, the four teams we will recruit from specifically provide services for people with psychosis. In the other, the three teams work with people with psychosis and other conditions such as depression and anxiety.

Stage 1 Development of the intervention

Working Group: A working group was set up of five people including: members of the research team; a representative from each of the other network mapping projects i.e. a community navigator from Hounslow and the PI of the Community Navigator Study; and a GP working in one of the boroughs we will recruit from. The working group met during the development phase of the study.

Key Informant Interviews: In each site key informant interviews were held with primary care mental health leads and peer support service managers to identify how the intervention would link with other provision and any clinical governance arrangements to be put in place. The PI liaised with mental health services through attendance at team meetings. The aim of this was to: introduce and generate a sense of ownership over the idea of the intervention; consult on the arrangements identified through the key informant interviews; discuss how to identify potential participants and agree the arrangements with any amendments needed.

Pre-pilot of the mapping process: A manual was drafted by the research team and PSWs who helped to refine and streamline the process. It was then tested by PSWs on each other and with the researcher. The research team observed and

For peer review only

documented this process; data were field notes, interview transcripts and workshop outputs.

During the development of the intervention, two goals for the service user to work towards were added. The first goal was to visit a primary care service for a physical health appointment; the second was to create a behaviour change with a view to improving physical health. The aim was for the peer to encourage service users to utilise the map when working towards these goals as well as supporting them to attend an appointment by offering to accompany them.

Theory of change

The development of the intervention will be informed by a theory of change model (figure 1). This will help to identify the desired impacts and outcomes and the mechanism by which these are achieved. To refine the theory of change for further testing, we will use a mixed methods approach, collating quantitative outcome measures (pre and post intervention), and qualitative interviews and focus groups with both participants and PSWs. We will also conduct weekly supervision with PSWs and observe some of the intervention sessions.

<Insert figure 1 here>

Figure 1. Preliminary Theory of Change model.

For peer review only

Stage 2 Piloting

Recruitment

A member of the research team will approach clinical staff at community-based mental health team centres. They will be informed about the research, the inclusion/exclusion criteria, and given an information script. This will provide clinical staff with details of the study to relay to possible participants. Staff will be asked to consider their entire caseload, which usually consists of up to 30 people, and to contact eligible service users as agreed with the Research Ethics Committee. When

clinical staff receive agreement from a service user, a meeting will be organised; during which, a researcher will provide a patient information sheet and answer any questions. Participants will be offered a period of time, usually at least 24 hours, to consider their involvement in the study according to IRAS guidance (35), before providing consent to participant in the study.

Sample size

The target sample size of 24 is based on the pragmatics of testing the feasibility of delivering this intervention to inform a larger scale study, therefore this sample size is appropriate (36).

Inclusion and exclusion criteria

The following inclusion criteria will be used: 1) approaching discharge from community-based mental health services, as this group are soon to cease contact with mental health professionals who encourage healthy behaviours and help seeking for physical health; 2) at risk of deterioration in physical health due to poor access to primary care; 3) demonstrated failure to organise/attend primary care appointments for co-morbid physical health difficulties; 4) aged 18 or over; 5) clinical diagnosis of severe mental illness (a Schizophrenia Spectrum Disorder or Bipolar I).

People will not be eligible to take part in the study if they: 1) lack capacity to provide informed consent; this will be assessed during the consent process as the ability to retain, weigh up and make decisions based on the information provided; 2) pose a high risk of harm to others, as judged by their clinical team; 3) are unable to communicate in English; 4) are currently an inpatient in a psychiatric hospital; and 5) nonattendance of discharge planning meeting with subsequent discharge.

Patient and public involvement

PSWs, who have lived experience of mental health problems, were involved in the development of the intervention through their provision of feedback on the draft

For peer review only

manual, and will deliver and feed back their views on the intervention. We also brought together a group of peer support workers and others involved in projects with the McPin Foundation that include a mapping component, as advisors. They helped refine the mapping process, contributed to the planned training programme and inputted into the draft ToC.

The intervention

Peer support workers: PSWs (n=6) were recruited from within existing peer support services in the two London boroughs (three per borough). The PSWs were paid to take on the peer well-being role. We aimed that each PSW will work with up to 4 clients. The training took place across two days. One day focused on the intervention, including the PSWs carrying out the mapping intervention with one another, and development of the manual. The second day was a refresher on the key aspects of peer support. Training was delivered by a network mapping researcher (DS), a researcher with expertise on peer support (SG), and a researcher who lead on intervention development with input from the whole study team (JD). PSWs will be supported with use of the well-being mapping tool through regular supervision with the research team and their line manager.

Over six sessions, the intervention aims to cover the following topics: engagement of the client with the peer and the intervention; mapping of the client's network (see figure 2 for an example map), including people and services which contribute positively, negatively, or both, to the client's well-being; goal setting for physical health; and planning a visit to a primary care service e.g. their GP's surgery, dentist, or sexual health clinic, to which the client may or may not be accompanied by the peer; monitoring of work towards the goals and review of the primary care visit; and an ending, to include a review and summary of the client's achievements, updating the well-being map and encouragement to continue to utilise it. The visit to a primary care service will be prepared for collaboratively using resources which aim to prevent diagnostic overshadowing. For example, topics to be discussed in the appointment will be pre-agreed between the PSW and service user.

<Insert figure 2 here>

For peer review only

Figure 2. Example of a network map.

Some people with SMI may not have robust or wide social networks. In these cases, the PSWs will make suggestions for ways to expand the person's social network. The observations, notes from supervision with PSWs, and qualitative feedback will identify whether additions to the intervention are needed to try to build social networks.

The network mapping process contributes to reflexivity, allowing participants to become more aware of the resources they have in networks to support physical health, and make positive behavioural change. Physical health can be activated indirectly through person-centred goals. A person might not be motivated to address physical health concerns, but they might be motivated to address an area of the well-being network map that matters to them. The peer can use these personal goals to indirectly improve the individual's physical health goals. For example, if they wish to join a book club to meet new people, the peer could suggest they walk/cycle to this club.

Participants can arrange to meet with their PSW in a location that suits them both. This may include private rooms at their community-based mental health service, a library or a café. The six sessions will in most cases take place across 6 weeks, however this time period can be elongated to reflect the needs of the service user and to allow a primary care visit. Each session will last up to two hours. The sessions are guided by the manual, however the map and the goals created will be personalised to the individual.

Baseline and follow up procedures

Baseline data collection will take place following consent and before the initial meeting with the PSW. Participants will meet with a PSW and begin the intervention. After finishing the intervention, participants will be asked to complete outcome measures at four months from baseline, before completing a semi structured qualitative interview. PSWs will also take part in a semi structured qualitative interview and a focus group. The qualitative interviews will document the participant's view of their physical health and its management; experiences of the

For peer review only

intervention; ways in which it worked; identify mechanisms through which the mapping helped lead people to take action on their physical health; and factors which limited its helpfulness; and how it could be improved. A focus group with all PSWs across both sites will seek feedback on use of the mapping tool; discuss the actions planned versus those taken, and reasons for deviation; problems encountered and methods for solving them; and the outcomes achieved.

Participants will be compensated £10 for each of their baseline and follow up assessments and interviews. A further £15 will be given for focus group participation.

Outcomes

The peer support change model developed by Gillard at al (37) was used to identify outcomes and hence, the following outcomes will be measured pre intervention and at a 4 month follow up after the intervention has been completed:

Service use and demographic information (age, sex, marital status, ethnicity, nationality, language, education, employment and housing status) will be measured using an adapted version of the Client Service Receipt Inventory (38). Health and social care, informal care and use of leisure activities were measured. Primary care and secondary care (for physical health care) will be used as intermediate outcomes on the pathway to improved physical health; likewise leisure activities with potential benefit for physical health such as those involving exercise.

Social capital will be measured using the Resource Generator UK (39) (RG-UK). The RG-UK asks participants if they have or could obtain access to each of 27 skills or resources within their social network within one week. It then asks the nature of the social tie through which they could access each skill or resource. The instrument has four sub-scales: domestic resources, personal skills, expert advice and problem solving resources. This measure assesses service use and healthy behaviours to capture improvements to management of physical health. It reflects reciprocal relationships, so change may be due to either the participant, members of their network, or both. It has good reliability and validity, and has been used in samples of people with mental health problems e.g., producing valid results. The RG-UK does not fully measure access to resources related to health, the primary interest of this

For peer review only

study. Consequently an additional subscale for the RG-UK was used, developed by Pinfold et al (40), which captures health and well-being related resources.

Social network quality and quantity will be measured using the Lubben Social Network Scale is a 6-item self-report measure assessing quantity and quality of contact with family and friends (41). Three questions to capture acquaintances were added to this measure.

Hope will be measured using the Herth Hope Index (42), a 12 item self-report measure designed to measure levels of hope in adults in clinical settings, increasingly used in mental health studies. This measure has been found to hold divergent and construct validity(42).

Mental health self-efficacy will be measured using the Mental Health Confidence Scale (43), an 11 item self-report measure based on theories of self-efficacy and self-help. The scale was shown to have good internal consistency and clinical utility (44).

Mental well-being will be measured using the Short Warwick-Edinburgh Mental Well-being Scale (SWEMWBS) (45), a 7-item measure designed to measure mental well-being, found to have good reliability and validity for people with SMI (46). The positively worded questions focus on assessing mental well-being as opposed to mental health difficulties.

Physical health will be measured using the EuroQol Five Dimensions Questionnaire (47), a two-part questionnaire assessing the objective and subjective health status of participants. The measure can be used to compute a Quality Adjusted Life Year (QALY) (48), an outcome frequently used in cost analyses, and has been found to be a valid measure among people with schizophrenia (49).

Some outcomes will not be measured, such as: emotions relating to unhealthy behaviours; health beliefs; self-identify; social norms; or behaviours of friends and family. These are intermediate outcomes and we have prioritised outcomes further along the ToC model, to reduce respondent burden.

Process evaluation

For peer review only

With the permission of both PSW and participant, the project researcher will observe PSWs delivering some sessions of the intervention to participants in vivo. The aim of this observation is to evaluate the extent of the use of the mapping tool; whether and if so how it is used to inform goal setting and planning; and whether its use creates any difficulties in goal setting and planning. We will record information provided during supervision sessions between the research team and the PSWs.

Proposed data analysis

Quantitative data: Feasibility of the intervention will be assessed by rates of recruitment of peers and of clients at each site; implementation of the intervention – i.e. what proportions of participants had each of 0-6 sessions with a peer and what proportion visited their GP or other primary care service for a physical health issue. Feasibility of the evaluation will be assessed by completion rates of interviews and measures of peers and clients; rates of attendance at focus groups. Floor to ceiling effects of measures will be identified, as well as the level of outcome measure completion.

Qualitative data: Data from the first two participants at each site will be rapidly analysed by the study researchers with the main aim of identifying any feasible modifications needed to the intervention or study design. We will use a focussed thematic analysis approach (50) to identify: contextual barriers and facilitating factors to the successful implementation of the intervention with respect to the desired outcomes; the appropriateness of the selected outcomes (above); and mechanisms of action of the intervention. Multiple coding will be conducted on five transcripts to allow researchers to identify and discuss any alternative interpretations. The study researcher will input all coding using NVivo, with cross checks by another team member. We will hold analysis meetings to review transcripts and develop coding frames. Further synthesis meetings will be required once coding is complete.

Stage 3. Collated feedback

For peer review only

Stakeholders from both boroughs will be brought together to discuss the results. The outputs following the workshop will include: 1) agreed documentation for use including guides for both PSWs and clients to support the model; 2) select and refine outcomes for a randomised controlled trial; 3) manuals to document the well-being mapping process – one for clients and one for well-being partners.

Ethics and dissemination

Ethics approval for this study has been obtained from the Heath and Research Authority (HRA) and the London- Chelsea Regional Ethics Committee (REC); ref 17/LO/0585.

We plan to disseminate the results to the participants who received the intervention and the PSWs who delivered the intervention. We will also share the results with the NHS trusts we recruited from, the peer support services from which the PSWs were recruited, primary care mental health leads and commissioners. We plan to publish the findings in peer-reviewed journals and share our findings at academic conferences.

REFERENCE LIST

1. Wahlbeck K, Westman J, Nordentoft M, Gissler M, Laursen TM. Outcomes of Nordic mental health systems: life expectancy of patients with mental disorders. British Journal of Psychiatry. 2011.

2. Osborn DPJ, Nazareth I, King MB. Physical activity, dietary habits and Coronary Heart Disease risk factor knowledge amongst people with severe mental illness. Social Psychiatry and Psychiatric Epidemiology. 2007;42(10):787-93.

3. Matcham F, Carroll A, Chung N, Crawford V, Galloway J, Hames A, et al. Smoking and common mental disorders in patients with chronic conditions: an analysis of data collected via a web-based screening system. General hospital psychiatry. 2017;45:12-8.

4. John A, McGregor J, Jones I, Lee SC, Walters JT, Owen MJ, et al. Premature mortality among people with severe mental illness—New evidence from linked primary care data. 2018.

For peer review only

5. Reilly S, Planner C, Hann M, Reeves D, Nazareth I, Lester H. The role of primary care in service provision for people with severe mental illness in the United Kingdom. PLoS One. 2012;7(5):e36468.

6. Gillard S. The redistribution of mental health care: good, bad or just a bit ugly? J Ment Health. 2015;24(6):339-41.

7. Phelan M, Stradins L, Morrison S. Physical health of people with severe mental illness: can be improved if primary care and mental health professionals pay attention to it. BMJ: British Medical Journal. 2001;322(7284):443.

8. Bittles AH, Bev A. Petterson, Sheena G. Sullivan, Rafat Hussain, Emma J. Glasson, and Philip D. Montgomery. The influence of intellectual disability on life expectancy. The Journals of Gerontology Series A: Biological Sciences and Medical Sciences. 2002;57(7):M470 - M2.

9. Lester H, Tritter JQ, Sorohan H. Patients' and health professionals' views on primary care for people with serious mental illness: focus group study. BMJ. 2005;330(7500):1122.

10. Bindman J, Johnson S, Wright S, Szmukler G, Bebbington P, Kuipers E, et al. Integration between primary and secondary services in the care of the severely mentally ill: patients' and general practitioners' views. BrJPsychiatry. 1997;171:169-74.

11. Burns T, Greenwood, N., Kendrick, T., Garland, C. Attitudes of general practitioners and community mental health team staff towards the locus of care for people with chronic psychotic disorders. Int J Psychiatry Clin Pract. 2000;6:67-71.

12. Lester H, Gask L. Delivering medical care for patients with serious mental illness or promoting a collaborative model of recovery? BrJPsychiatry. 2006;188:401-2.

13. Corrigan PW, Mittal D, Reaves CM, Haynes TF, Han X, Morris S, et al. Mental health stigma and primary health care decisions. Psychiatry Res. 2014;218(1-2):35-8.

14. Graber MA, Bergus G, Dawson JD, Wood GB, Levy BT, Levin I. Effect of a patient's psychiatric history on physicians' estimation of probability of disease. Journal of General Internal Medicine. 2000;15:204-6.

15. Clarke DE, Dusome D, Hughes L. Emergency department from the mental health client's perspective. International Journal of Mental Health Nursing. 2007;16:126-31.

For peer review only

16. Jones S, Howard L, Thornicroft G. 'Diagnostic overshadowing': worse physical health care for people with mental illness. Acta Psychiatrica Scandinavica. 2008;118(3):169-71.
17. van Nieuwenhuizen A, Henderson C, Kassam A, Graham T, Murray J, Howard LM, et al. Emergency Department staff views and experiences on diagnostic overshadowing related to people with mental illness Epidemiology and Psychiatric Sciences. 2013;22(3):255-62.
18. Peckover S, Chidlaw RG. Too frightened to care? Accounts by district nurses working with clients who misuse substances. Health Soc Care Community. 2007;15(3):238-45.
19. Roberts L, Roalfe A, Wilson S, Lester H. Physical health care of patients with schizophrenia in primary care: a comparative study. Family Practice. 2007;24(1):34-40.
20. Pitt V, Lowe D, Hill S, Prictor M, Hetrick SE, Ryan R, et al. Consumer-providers of care for adult clients of statutory mental health services. Cochrane Database Syst Rev. 2013;3:CD004807.
21. Gillard S, Holley J, Gibson S, Larsen J, Lucock M, Oborn E, et al. Introducing New Peer Worker Roles into Mental Health Services in England: Comparative Case Study Research Across a Range of Organisational Contexts. 2015;42(6):682-94.
22. Moran GS, Russinova Z, Gidugu V, Yim JY, Sprague CJQHR. Benefits and mechanisms of recovery among peer providers with psychiatric illnesses. 2012;22(3):304-19.
23. Bellamy C, Schmutte T, Davidson L. An update on the growing evidence base for peer support. 2017;21(3):161-7.
24. Cabassa LJ, Camacho D, Vélez-Grau CM, Stefancic AJJopr. Peer-based health interventions for people with serious mental illness: a systematic literature review. 2017;84:80-9.
25. Kelly E, Duan L, Cohen H, Kiger H, Pancake L, Brekke J. Integrating behavioral healthcare for individuals with serious mental illness: A randomized controlled trial of a peer health navigator intervention. Schizophr Res. 2016.
26. Druss BG, Zhao L, von Esenwein SA, Bona JR, Fricks L, Jenkins-Tucker S, et al. The Health and Recovery Peer (HARP) Program: a peer-led intervention to improve medical self-management for persons with serious mental illness. Schizophr Res. 2010;118(1-3):264-70.

For peer review only

27. Goldberg RW, Dickerson F, Lucksted A, Brown CH, Weber E, Tenhula WN, et al. Living well: an intervention to improve self-management of medical illness for individuals with serious mental illness. Psychiatr Serv. 2013;64(1):51-7.
28. Pinfold V, Sweet, D. Wellbeing networks and asset mapping. http://mcpin.org/wp-content/uploads/wellbeing-networks-SW-VERSION.pdf 2015 [
29. Sweet D, Byng R, Webber M, Enki DG, Porter I, Larsen J, et al. Personal well-being networks, social capital and severe mental illness: exploratory study. The British Journal of Psychiatry. 2017.
30. Lloyd-Evans B, Bone JK, Pinfold V, Lewis G, Billings J, Frerichs J, et al. The Community Navigator Study: a feasibility randomised controlled trial of an intervention to increase community connections and reduce loneliness for people with complex anxiety or depression. 2017;18(1):493.
31. Webber M, Reidy H, Ansari D, Stevens M, Morris DJRoSWP. Developing and modeling complex social interventions: Introducing the connecting people intervention. 2016;26(1):14-9.
32. Foundation TM. Wellbeing Networks and Mental Health: The McPin Foundation; 2018 [Available from: http://mcpin.org/networks-and-mental-health/.
33. Kail A, Lumley, T. The theory of change – the beginning of making a difference. . London: New Philanthropy Capital; 2012.
34. Harris E, Hodgson, L., Noble, J. Creating your theory of change – NPC's practical guide. http://www.thinknpc.org/publications/creating-your-theory-of-change/: NPC; 2014
35. Integrated Research Application System (IRAS). Collated Question-Specific Guidance for NHS REC Form; 2018 [Available from: https://www.myresearchproject.org.uk/help/hlpcollatedqsg-nhsrec.aspx]
36. Leon AC, Davis LL, Kraemer HC. The role and interpretation of pilot studies in clinical research. Journal of psychiatric research. 2011;45(5):626-9.
37. Gillard S, Gibson SL, Holley J, Lucock M. Developing a change model for peer worker interventions in mental health services: a qualitative research study. Epidemiol Psychiatr Sci. 2015;24(5):435-45.
38. Beecham J, Knapp M. Costing psychiatric interventions. In: Thornicroft G, Brewin CR, Wing JK, editors. Measuring Mental Health Needs. London: Royal College of Psychiatrists, Gaskell; 1992. p. 34-47.

For peer review only

39. Webber M, Corker E, Hamilton S, Weeks C, Pinfold V, Rose D, et al. Discrimination against people with severe mental illness and their access to social capital: findings from the Viewpoint survey. EpidemiolPsychiatrSci. 2013.

40. Vanessa Pinfold DS, Ian Porter, Cath Quinn, Richard Byng, Chris Griffiths, Julie Billsborough, Doyo Gragn Enki, Ruth Chandler, Martin Webber, John Larsen, John Carpenter, and Peter Huxley. Improving community health networks for people with severe mental illness: a case study investigation. Health Services and Delivery Research. 2015;3(5).

41. Lubben J, Blozik E, Gillmann G, Iliffe S, von Renteln Kruse W, Beck JC, et al. Performance of an abbreviated version of the Lubben Social Network Scale among three European community-dwelling older adult populations. Gerontologist. 2006;46(4):503-13.

42. Herth K. Abbreviated instrument to measure hope: development and psychometric evaluation. J Adv Nurs. 1992;17(10):1251-9.

43. Carpinello SK, EL; Markowitz, FE; Pease, EA. The development of the Mental Health Confidence Scale: a measure of self-efficacy in individuals diagnosed with mental disorders. Psychiatric Rehabilitation Journal. 2000;23(3):236-43.

44. Castelein S, van der Gaag M, Bruggeman R, van Busschbach JT, Wiersma DJPS. Measuring empowerment among people with psychotic disorders: a comparison of three instruments. 2008;59(11):1338-42.

45. Tennant R, Hiller L, Fishwick R, Platt S, Joseph S, Weich S, et al. The Warwick-Edinburgh mental well-being scale (WEMWBS): development and UK validation. Health and Quality of Life Outcomes. 2007;5.

46. Vaingankar JA, Abdin E, Chong SA, Sambasivam R, Seow E, Jeyagurunathan A, et al. Psychometric properties of the short Warwick Edinburgh mental well-being scale (SWEMWBS) in service users with schizophrenia, depression and anxiety spectrum disorders. 2017;15(1):153.

47. Williams A. Euroqol - a New Facility for the Measurement of Health-Related Quality-of-Life. Health Policy. 1990;16(3):199-208.

48. Fanshel S, Bush JW. Health-Status Index and Its Application to Health-Services Outcomes. Operations Research. 1970;18(6):1021-66.

49. Mulhern B, Mukuria C, Barkham M, Knapp M, Byford S, Brazier JJTBJoP. Using generic preference-based measures in mental health: psychometric validity of the EQ-5D and SF-6D. 2014;205(3):236-43.

For peer review only

50. Braun VaC, V. Using thematic analysis in psychology. Qualitative Research in Psychology. 2006;3(2):77 - 101.

Data statement

The datasets used and/or analysed during the current study are available from the corresponding author on reasonable request.

Authors Statement

JC was the research assistant responsible for recruitment of participants to the study, data collection and peer supervision. JD was the research assistant responsible for helping develop the intervention, train the peer support workers and recruited participants to the study. CH was the chief investigator. DS and VP helped to develop the intervention. DS helped to train the peers. SG was the site lead and helped to train peers. JC drafted the paper and all authors provided edits and comments for its revision.

Acknowledgements

Bryn Lloyd-Evans, Amanda Perry, Sally Gomme (Hounslow), Nancy Kuchemann (GP and Southwark CCG clinical lead) and the Southwark and Sutton peer support services.

Funding

This research was supported by the Maudsley Charity and the National Institute for Health Research (NIHR) Collaboration for Leadership in Applied Health Research and Care South London (NIHR CLAHR South London) at King's College Hospital NHS Foundation Trust. The views expressed in this article are of those of the authors and not necessarily those of the NHS, the NIHR or the Department of Health and Social Care.

Conflicts of Interest

The authors declare that they have no competing interests.

For peer review only

For peer review only

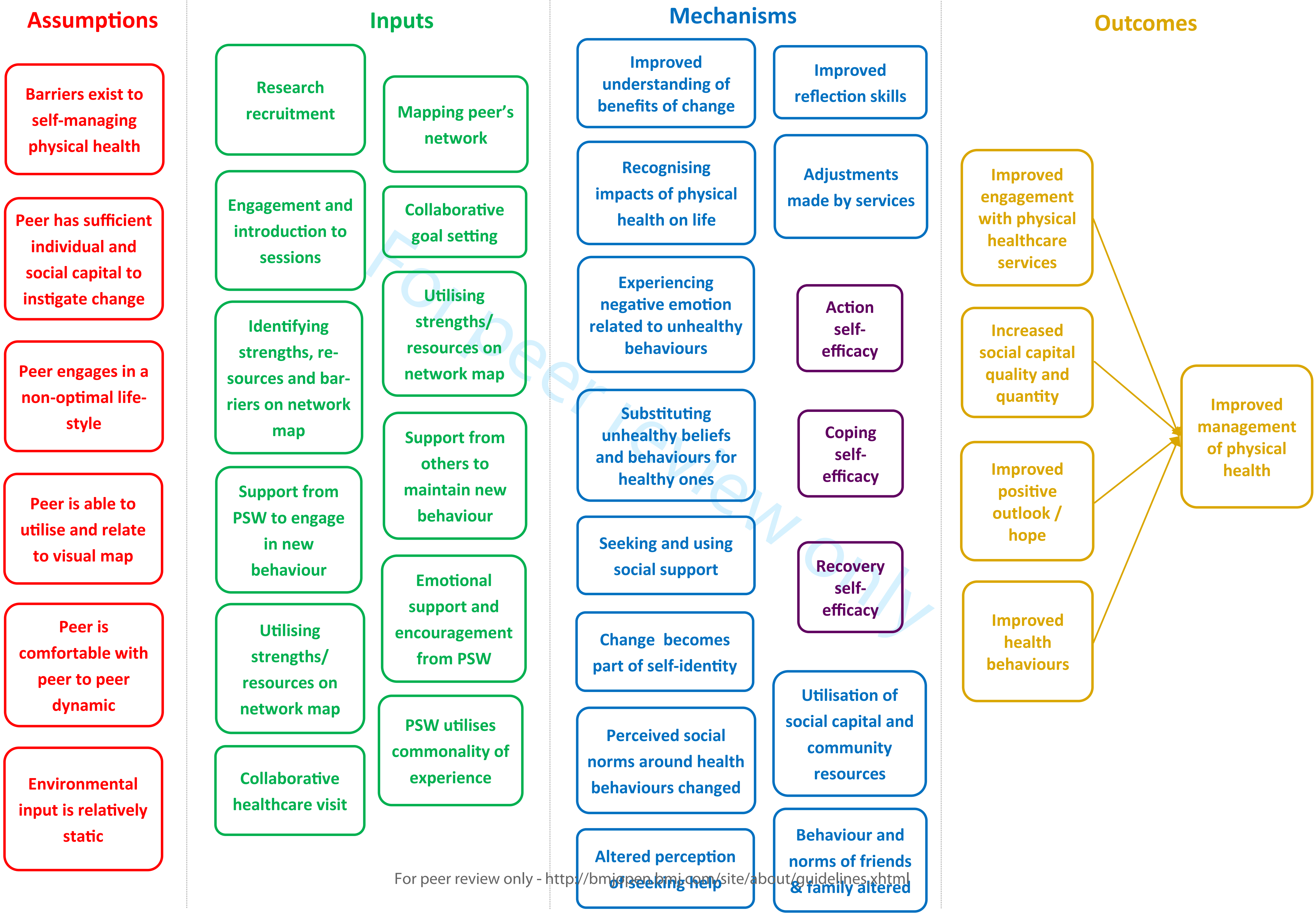
Assumptions
Barriers exist to self-managing physical health
Peer has sufficient individual and social capital to instigate change
Peer engages in a non-optimal life-style
Peer is able to utilise and relate to visual map
Peer is comfortable with peer to peer dynamic
Environmental input is relatively static
Inputs
Research recruitment
Mapping peer's network
Engagement and introduction to sessions
Collaborative goal setting
Identifying strengths, re-sources and bar-riers on network map
Utilising strengths/ resources on network map
Support from others to maintain new behaviour
Support from PSW to engage in new behaviour
Emotional support and encouragement from PSW
Utilising strengths/ resources on network map
PSW utilises commonality of experience
Collaborative healthcare visit
Mechanisms
Improved understanding of benefits of change
Improved reflection skills
Recognising impacts of physical health on life
Adjustments made by services
Experiencing negative emotion related to unhealthy behaviours
Action self-efficacy
Substituting unhealthy beliefs and behaviours for healthy ones
Coping self-efficacy
Seeking and using social support
Recovery self-efficacy
Change becomes part of self-identity
Utilisation of social capital and community resources
Perceived social norms around health behaviours changed
Altered perception of seeking help
Behaviour and norms of friends & family altered
Outcomes
Improved engagement with physical healthcare services
Increased social capital quality and quantity
Improved positive outlook / hope
Improved health behaviours
Improved management of physical health

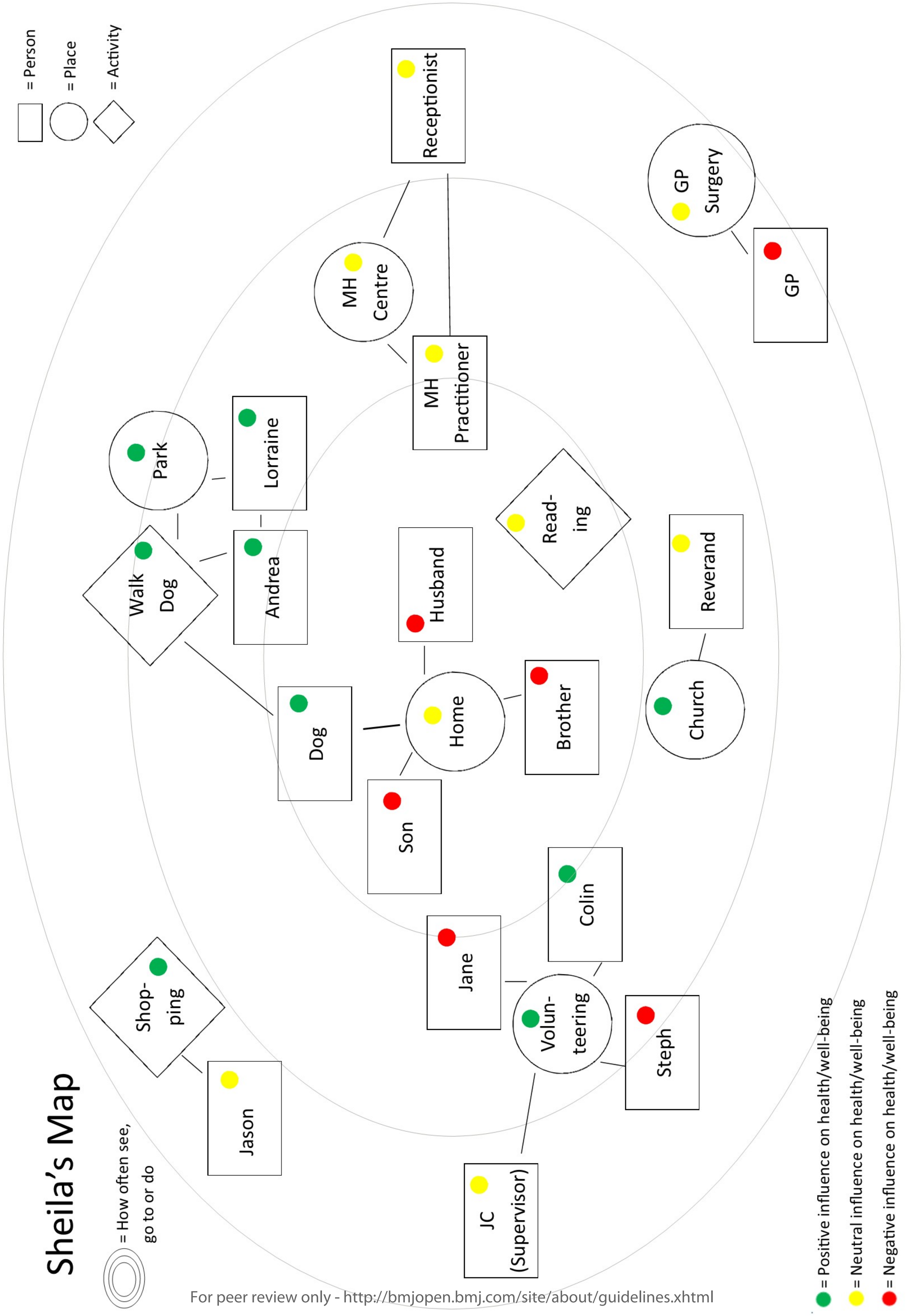
Sheila's Map
= Person
= Place
= Activity
= How often see, go to or do
= Positive influence on health/well-being
= Neutral influence on health/well-being
= Negative influence on health/well-being
Receptionist
GP Surgery
GP
MH Centre
MH Practitioner
Park
Lorraine
Walk Dog
Andrea
Read-ing
Husband
Reverand
Home
Dog
Brother
Church
Son
Colin
Jane
Volun-teering
Steph
Shop-ping
Jason
JC (Supervisor)
